# Instrument–Software Synergy in Proteomics: Systematic Evaluation across Mass Spectrometry Platforms, Search Engines, and Rescoring Methods

**DOI:** 10.1021/acs.jproteome.6c00295

**Published:** 2026-07-14

**Authors:** Sander Heyndrickx, Robbin Bouwmeester, Arthur Declercq, Robbe Devreese, Magnus Palmblad, Wout Bittremieux, Tess AV Afanasyeva, Joel Lapin, Arzu Tugce Guler, Pierre-Olivier Schmit, Christine Carapito, Lennart Martens, Ralf Gabriels

**Affiliations:** † 82219 CompOmics, VIB-UGent Center for Medical Biotechnology, VIB, Ghent 9000, Belgium; ‡ Department of Biomolecular Medicine, Faculty of Medicine and Health Sciences, Ghent University, Ghent 9000, Belgium; § Center for Proteomics and Metabolomics, 4501 Leiden University Medical Center, Postbus 9600 2300 RC Leiden, The Netherlands; ∥ Department of Computer Science, 129065 University of Antwerp, Antwerpen 2020, Belgium; ⊥ Sanquin Research and Landsteiner Laboratory, 1234 Amsterdam UMC, University of Amsterdam, Amsterdam 1066 CX, Netherlands; ∇ Computational Mass Spectrometry, TUM School of Life Sciences, Technical University of Munich, Freising 85354, Germany; ○ Institute for Experiential AI, 1848 Northeastern University, Boston, Massachusetts 02115, United States; ◆ Department of Pathology, Boston Children’s Hospital, Boston, Massachusetts 02115, United States; ¶ Harvard Medical School, Boston, Massachusetts 02115, United States; △ Bruker France SAS, Wissembourg 67160, France; ▲ BioOrganic Mass Spectrometry Laboratory (LSMBO), IPHC UMR 7178, University of Strasbourg, CNRS, Strasbourg 67000, France; ▽ Infrastructure Nationale de Protéomique ProFI − UAR 2048, Strasbourg 67087, France

**Keywords:** mass spectrometry, proteomics, instruments, data analysis, peptide identification, peptide quantification, machine learning, evaluation

## Abstract

Mass spectrometry-based proteomics has advanced through parallel improvements in instrumentation (mass spectrometers) and software (data analysis), yet whether these improvements interact synergistically or provide diminishing returns remains unclear. Here, we systematically evaluate instrument–software coevolution across eight mass spectrometry platforms, three generations of search engines, and multiple rescoring approaches, yielding 72 unique instrument–software combinations spanning from 2004 to 2024. Our results reveal that instrumentation and software improvements produce synergistic rather than substitutive benefits. Here, machine learning-based rescoring consistently recovers identifications from low-intensity precursors that produce noisier, more challenging spectra. Crucially, because of increased sensitivity and speed, modern instruments detect more low-intensity precursors, thereby increasing the population of challenging spectra for which rescoring provides the greatest benefit. However, this expanded detection depth comes at a cost: recovered low-abundance peptides exhibit inherently higher quantification error, creating a fundamental trade-off between proteome coverage and quantification accuracy. Together, these findings provide a systematic overview of how instrument and software advances have jointly shaped proteomics performance over the past two decades.

## Graphical abstract

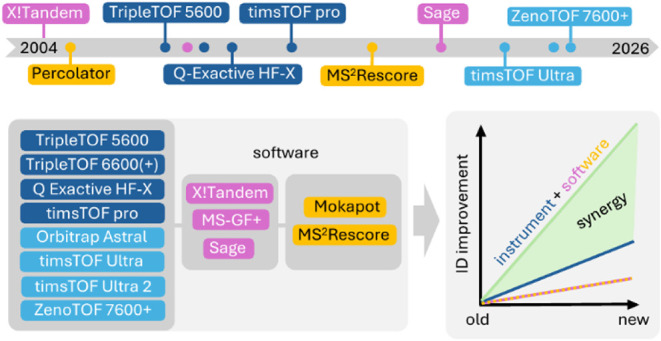

## 1 Introduction

Liquid chromatography coupled to tandem mass spectrometry (LC-MS/MS) is the cornerstone of bottom-up proteomics, enabling researchers to identify and quantify thousands of proteins from complex biological and environmental samples.^1^ The experimental workflow follows a well-established pipeline.^2^ Proteins are enzymatically digested into peptides, which are then separated by liquid chromatography, ionized by electrospray ionization, and fragmented, most commonly through collision-induced dissociation. Specialized search engines compare observed spectra against theoretical fragmentation patterns from sequence databases, producing peptide-spectrum matches (PSMs) that are typically filtered at a 1% false discovery rate (FDR).^3^

While core concepts have remained the same, this workflow has seen vast improvements over the years. First, at the instrument layer, mass spectrometry instruments have evolved dramatically. Each generation improved upon the previous generation with better precursor isolation, ion manipulation, mass accuracy, resolution, and speed. For example, MS/MS acquisition rates increased from typically 20 Hz on the TripleTOF 5600 to over 300 Hz with PASEF on the timsTOF Ultra 2, while the addition of trapped ion mobility spectrometry introduced an entirely new dimension of separation. Second, at the search engine layer, computational analysis has undergone its own evolution. Search engines have progressed from early tools like SEQUEST,^4^ Mascot,^5^ and X!Tandem^6^ through MS-GF+^7^ to modern implementations like Sage,^8^ each incorporating algorithmic innovations that improve scoring accuracy and computational efficiency.^9^ Third, at the postprocessing layer, rescoring methods have substantially improved identification rates. Tools like Percolator^10^ and Mokapot^11^ employ semisupervised learning to learn discriminative boundaries from PSM features. This more effectively separates true positives from false positives compared to a static score used in most search engines. Rescoring confidently identifies PSMs that would otherwise fall below the search engine’s FDR threshold. More recently, the integration of machine learning (ML)-based peptide property predictors has further enhanced this process by providing additional orthogonal layers of evidence for rescoring.^12–14^ These tools predict properties such as retention time and fragment ion intensities, which are compared against experimental observations to produce similarity metrics that serve as new features for the rescoring model. The resulting increase in identification rates has made ML-based rescoring a standard component of many modern proteomics pipelines, with automated frameworks like MS^2^ Rescore^159^, AlphaPeptDeep,^16^ Oktoberfest,^17^ and MSBooster^18^ widely adopted by the community.

Despite parallel advances in instrumentation and computational analysis, their combined impact and interaction have never been systematically quantified. It remains unclear whether instrument and software improvements interact synergistically, independently, or exhibit diminishing returns. On one hand, higher quality spectra with more consistent fragmentation patterns might provide richer information for ML to exploit, amplifying its benefit. Alternatively, modern instruments may already measure spectra of such high quality that more advanced software offers only marginal improvements, suggesting that as instrumentation continues to advance, the value of sophisticated software diminishes.

Here, we present a systematic evaluation that jointly characterizes both instrument and software evolution in data-dependent acquisition (DDA) proteomics workflows. We focus on DDA because it offers the longest historical record across instrument and software generations. While our evaluation is restricted to DDA, we expect the general findings on instrument–software synergy to extend to data-independent acquisition, as discussed in Section 4.5. Within this scope, we examine eight mass spectrometry platforms commonly used in proteomics research, combined with three generations of search engines (X!Tandem,^6^ MS-GF+,^7^ and Sage^8^) and three rescoring conditions (no rescoring, Mokapot,^11^ and MS^2^ Rescore^15^). These 72 unique instrument–software combinations allow us to address a central question: do instrument and software advances produce independent, diminishing, or compounding benefits—and, if so, where in the proteomics workflow are these interactions most consequential? We provide the first structured evaluation of technological coevolution in proteomics by decomposing identification, sensitivity, coverage, diversity, and quantification performance across 72 configurations.

## 2 Material and Methods

### 2.1 Data Availability

The raw mass spectrometry data used in this study were previously deposited in the PRIDE Archive via ProteomeXchange with identifiers PXD028735 and PXD070049. The analysis pipeline source code, configuration files, processed results, and all intermediate outputs generated in this study are available on Zenodo (https://zenodo.org/records/18743931). The Docker image tags and software versions used in the analysis pipeline are listed in Supporting Information Tables 2 and 3, respectively. This research did not involve human or animal participants.

### 2.2 Experimental Data

To systematically evaluate the interplay between instrument and software evolution in proteomics, we utilized the comprehensive label-free quantification benchmark data sets published by Van Puyvelde et al.^19,20^ (PXD028735 and PXD070049). This combined data set provides an ideal resource for cross-platform comparison, as it was specifically designed to assess data analysis pipelines across multiple instrument generations using a standardized experimental protocol. The consistent sample preparation and acquisition parameters across all instruments and batches enable a direct comparison of performance metrics while isolating the specific contributions of instrumentation and software components.

The benchmark consists of a three-species protein mixture: *Homo sapiens* (human), *Saccharomyces cerevisiae* (yeast), and *Escherichia coli*, combined in different ratios: 65% human, 30% yeast, and 5% *E. coli* for condition α, and 65% human, 15% yeast, and 20% *E. coli* for condition β (Figure 1). The data set includes three technical replicates for conditions α and β acquired across multiple mass spectrometry platforms spanning over a decade of technological advancement (Table 1). Notably, sample load and gradients differ between instruments (Table 1). This reflects standard operating practices for each platform. Chromatographic setups also differed (see Supplementary Table 1), which we acknowledge as an unaccounted source of variation in this comparison.

**Table 1:** Mass Spectrometry Platforms and Acquisition Parameters Used in This Study^a^

**instrument**	**year**	**data source**	**sample load**	**gradient (min)**
SCIEX TripleTOF 5600	2010	PXD028735	5 μg	120
SCIEX TripleTOF 6600(+)	2014	PXD028735	5 μg	120
Thermo Fisher Orbitrap Q-Exactive HF-X	2017	PXD028735	1 μg	120
Bruker timsTOF Pro	2018	PXD028735	1 μg	120
Thermo Fisher Orbitrap Astral	2023	PXD070049	50 ng, 250 pg	15, 5
Bruker timsTOF Ultra	2023	PXD070049	250 pg	15, 5
Bruker timsTOF Ultra 2	2024	PXD070049	50 ng	15, 5
SCIEX ZenoTOF 7600+	2024	PXD070049	50 ng	15

a Instruments are listed chronologically by commercial release year, spanning 2010–2024. Data Source indicates the PRIDE Archive identifier of each dataset. Note: the TripleTOF 6600+ (2019) is positioned at 2014 because its DDA instrumentation remains largely unchanged from the original 6600; “Plus” upgrades primarily introduced DIA-specific Scanning SWATH and a new ion source, which was mainly an ease-of-use upgrade.

**Figure 1:**
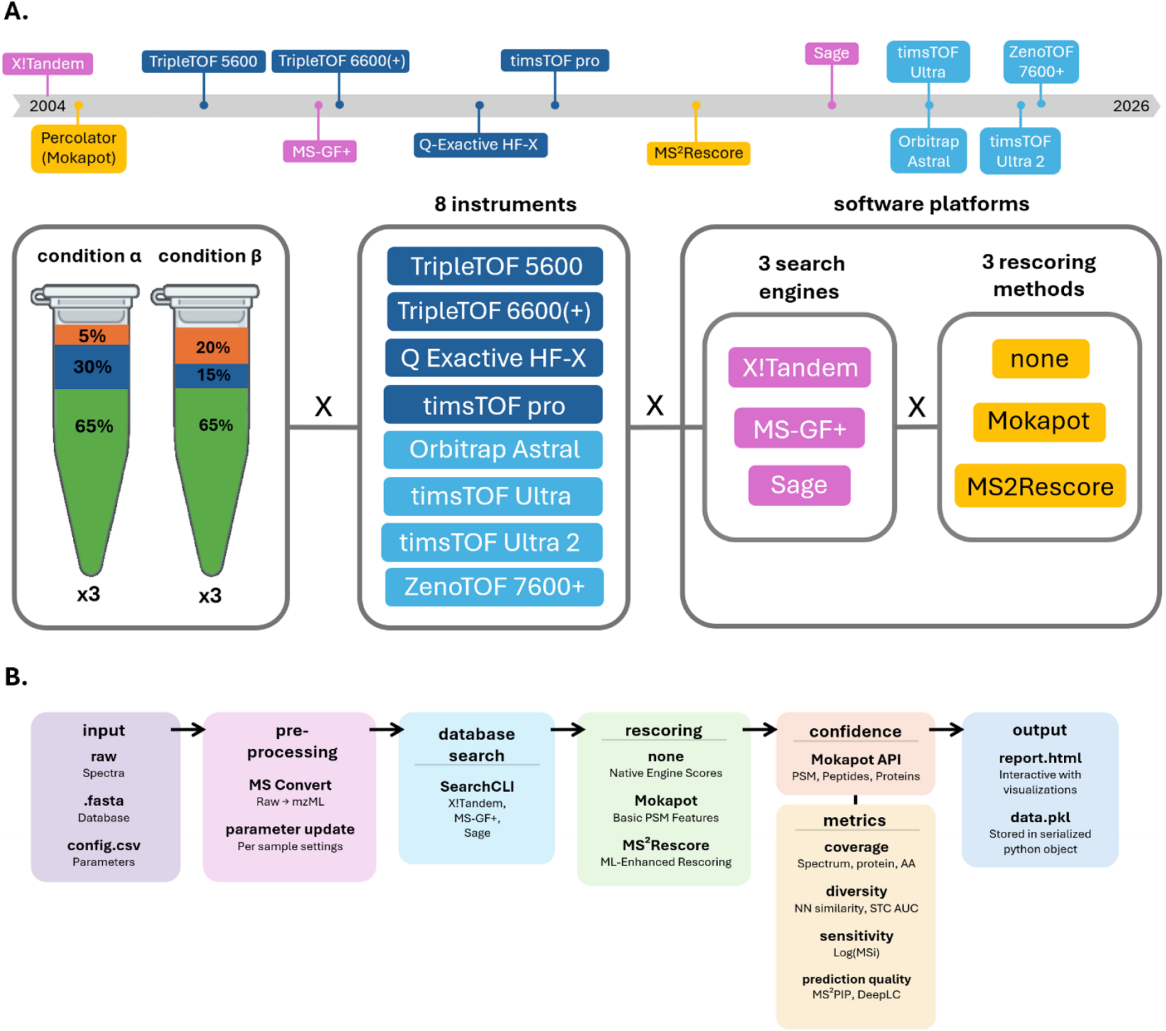
Experimental design for systematic evaluation of instrument–software coevolution in proteomics. (A) Timeline shows the release years of instruments (blue), key search engines (pink), and rescoring methods (yellow) spanning 2004–2026. Each of the two conditions (α and β, differing in yeast and *E. coli* ratios) was measured in triplicate across eight mass spectrometry platforms representing different instrument generations. Instruments shown in lighter colors were analyzed using low sample loads and short gradients. Each data set was processed through three search engines (X!Tandem, MS-GF+, Sage) spanning almost two decades of algorithmic development, with each search engine output analyzed under three postprocessing conditions: none (no rescoring), Mokapot (conventional PSM feature-based rescoring), and MS^2^ Rescore (ML-enhanced rescoring). (B) Overview of database searching and rescoring pipeline. Each raw mass spectrometry file is independently processed through five sequential stages. Preprocessing converts vendor formats to mzML and loads run-specific parameters from the configuration file. Database searching uses SearchCLI to execute all three search engines (X!Tandem, MS-GF+, Sage) against a target-decoy database. Each search engine output is processed under three rescoring conditions: none (native engine scores, no rescoring), Mokapot (conventional PSM feature-based rescoring), and MS^2^ Rescore (ML-enhanced rescoring). Confidence assignment applies Mokapot’s unified statistical framework to calculate *q*-values at PSM, peptide, and protein levels. Performance metrics quantify identification coverage, sequence diversity, sensitivity, and ML prediction accuracy. The pipeline generates interactive HTML reports and serialized Python objects for each run, enabling systematic comparison across instruments and software configurations.

### 2.3 Database Search and Rescoring Pipeline

We developed an automated and reusable pipeline that evaluates performance across the complete proteomics technology stack. The pipeline processes raw mass spectrometry data through three complementary layers: database searching using three generations of search engines spanning nearly two decades (X!Tandem: 2004, MS-GF+: 2014, Sage: 2023), postsearch rescoring using both conventional and ML-based features, and downstream analysis comparing identification rates, proteome coverage, and quantification precision. By combining eight instrument platforms with three search engines and three rescoring conditions, this design yields 72 unique instrument–software combinations, enabling systematic decomposition of how improvements at each layer contribute to overall performance.

The entire workflow (Sections 2.3.1–2.4) was implemented in Nextflow, with each tool executed within dedicated Docker containers to ensure reproducibility and portability across computing environments (all external Docker image tags can be found in Supporting Information Table 2). The pipeline processes each raw file independently to generate standardized performance metrics and identification results. The pipeline is configured through a simple CSV file specifying run-specific parameters for each data set.

#### 2.3.1 Raw File Conversion

The pipeline starts with converting raw mass spectrometry data to mzML format using MSConvert (ProteoWizard, version skyline_25.1.0.224).^21^ Because of limitations in vendor-specific format conversion on Linux systems, Bruker.d directories and SCIEX.wiff files were converted to mzML on a Windows machine prior to pipeline input. Exact conversion parameters for each vendor format are provided in Supporting Information Table 4.

#### 2.3.2 Database Searching

Peptide-spectrum matching was performed against a combined target-decoy protein sequence database constructed from the UniProtKB/Swiss-Prot canonical reference proteomes (downloaded 01/09/2025) of *Homo sapiens* (UP000005640, taxon ID 9606; 20,473 sequences), *Saccharomyces cerevisiae* strain S288C (UP000002311, taxon ID 559292; 6,067 sequences), and *Escherichia coli* K-12 (UP000000625, taxon ID 83333; 4,403 sequences). Isoforms were not included. This was supplemented with the common Repository of Adventitious Proteins (cRAP, 46 sequences) contaminant database. Decoy sequences were generated by reversing each target sequence using Mokapot’s^11^ decoy generation function.

To evaluate algorithmic evolution at the database search layer, we employed three open-source search engines spanning two decades of computational development. Open-source availability was a deliberate selection criterion, as it enables fully transparent, end-to-end reproducible benchmarking and integrates directly with the containerized Nextflow pipeline used in this study. The oldest search engine tested here, X!Tandem^6^ (v2015.12.15.2), relies on manually designed scoring functions grounded in domain expertise and empirical validation. Next, MS-GF+^7^ (v2024.03.26) introduced probabilistic scoring through its fragment ion intensity tables and *generating function* methodology. Finally, Sage^8^ (v0.14.6) combines fast candidate matching through sequence indexing with data-driven scoring via linear discriminant analysis, representing the current state-of-the-art performance in both speed and identification.

All three search engines were executed through SearchCLI (the command-line interface of SearchGUI^22^ (v4.3.15)). Searches were configured with trypsin digestion, allowing up to two missed cleavages, carbamidomethylation of cysteines as a fixed modification, and oxidation of methionine as a variable modification. Precursor and fragment mass tolerances were determined for each platform by first performing a wide-tolerance search with Sage and then selecting tolerances based on the observed mass error distributions of target and decoy PSMs. These platform-specific tolerances were subsequently applied to all three search engines.

#### 2.3.3 PSM Rescoring

To evaluate the contribution of postsearch processing, we applied two rescoring methods to each search engine’s output and compared their performance against native baselines—defined as PSM rankings derived solely from each search engine’s primary scoring function, without any postsearch rescoring. This experimental design allows us to assess how rescoring methods interact with both instrument platforms and initial search algorithms.

**Baseline**. For the baseline condition, raw search engine scores were used directly for FDR control without additional postprocessing.

**Conventional Rescoring**. Employed Mokapot^10,11^ (v0.10.0), which trains a linear support vector machine (SVM) with semisupervised learning to refine PSM discrimination using spectral features. These features were generated using the “basic feature generator” of MS^2^ Rescore^15^ (v3.1.5) (listed in Supplementary Table 5) and mainly served as a control to isolate the specific contribution of machine learning (ML)-derived features.

**ML-Enhanced Rescoring**. The second approach utilized MS^2^ Rescore to integrate ML-predicted peptide properties as additional discriminative features. This pipeline generated similarity scores between observed values and those predicted by MS^2^ PIP^23^ (v4.1.0) (fragment ion intensities) and DeepLC^24^ (v3.0.8) (retention times). For data acquired on ion mobility-enabled instruments (timsTOF Pro/Ultra 2), IM2Deep-^25^ predicted (v0.3.1) collisional cross sections (CCSs) provided an additional feature. This workflow represents the current state of the art in leveraging ML for postsearch refinement.

#### 2.3.4 Performance Assessment

##### 2.3.4.1 Confidence Assignment and Protein Inference

For consistency between different analysis workflows, the *q*-values were calculated with the *assign_confidence* function from the Mokapot Python API for all analysis conditions. This function uses the target-decoy approach to estimate *q*-values from either search engine scores (for baseline conditions) or rescored scores (for Mokapot and MS^2^Rescore conditions). This ensures that identification confidence is estimated in a consistent manner across all conditions, enabling direct comparisons among different software combinations.

The *assign_confidence* function operates hierarchically, computing *q*-values at three levels: peptide-spectrum matches (PSMs), peptides, and proteins. At the PSM level, *q*-values are estimated directly from score rankings. Peptide-level *q*-values were calculated by selecting the best PSM for each unique peptide sequence. Protein-level inference follows a parsimonious approach: proteins are ranked by their best supporting peptide, and *q*-values are assigned based on these rankings while accounting for shared peptides according to the picked-protein strategy implemented in Mokapot.^26^ All downstream analyses were performed using identifications filtered at a 1% FDR threshold at the PSM, peptide, and protein levels.

##### 2.3.4.2 Sensitivity Near the Detection Limit

To evaluate whether rescoring preferentially recovers identifications from low-abundance precursors, we required a per-spectrum measure of precursor signal strength at the moment of MS2 selection. Standard label-free quantification estimates peptide abundance by integrating the chromatographic elution profile, which conflates precursor abundance with peak shape, coelution effects, and the completeness of the extracted ion chromatogram. Instead, we estimated precursor abundance directly from the precursor ion intensity recorded in the single MS1 scan immediately preceding MS2 selection. Each precursor intensity was divided by the base peak intensity of its corresponding MS1 scan and log-transformed, yielding a normalized log-intensity value (log­(MSi)) for each spectrum. This scan-level normalization accounts for variation in total ion current between scans, enabling comparison of relative precursor abundance across the full acquisition. The resulting log­(MSi) distributions were then compared across rescoring conditions to quantify binwise improvements in identification rates at different intensity levels.

##### 2.3.4.3 Coverage

To evaluate the depth and breadth of proteome sampling, we calculated three complementary metrics: proteome coverage, proteome-wide sequence coverage, and the Gini coefficient of the coverage distribution.

**Proteome Coverage**. Represents the fraction of the reference proteome identified. This was calculated by dividing the number of identified target proteins (1% FDR, excluding decoys and contaminants) by the total number of entries in the sequence database. In cases of protein groups or peptides shared across species, every member was included, providing an optimistic estimate of the proteome-wide identification breadth.

**Proteome-Wide Sequence Coverage**. The coverage was calculated by mapping all confidently identified peptides to their positions in the reference proteome and dividing the total number of uniquely covered residues by the total number of amino acids in the reference database.

**Sampling Inequality**. The identified proteins were quantified by ranking identified proteins by their individual sequence coverage and plotting the cumulative proportion of total coverage against the cumulative proportion of proteins, yielding a Lorenz curve. From this, we extracted the Gini coefficient, which ranges from 0 (perfectly uniform) to 1 (highly skewed), where higher values indicate that sequence information is concentrated within a subset of the highly sampled proteins.

### 2.4 Cross-Run Comparative Analysis

#### 2.4.1 Label-Free Quantification Pipeline

To evaluate quantification accuracy and precision, we performed label-free quantification (LFQ) using FlashLFQ^27^ (v2.1.4). Due to current limitations in FlashLFQ’s compatibility with timsTOF data, quantitative analyses were restricted to non-Bruker instruments. For each instrument and search engine combination, peptide identifications from three technical replicates of conditions α and β were extracted from the primary analysis pipeline and used as input for quantification.

Downstream processing and normalization were performed using custom Python scripts, following similar methods to benchmarks in ProteoBench.^28^ To account for systematic run-to-run variation, peptide intensities were normalized by scaling each sample so that the median intensity of human peptides was equal across all runs, as the ratio of human peptides does not change between conditions.

Quantification performance was assessed by calculating the log_2_ fold-change (log_2_(mean_α_/mean_β_)) for each peptide. Here, mean_α_ and mean_β_ are the average (nonzero) peptide intensities across the 3 replicates. Accuracy was evaluated by determining the log_2_ fold-change error (observed minus expected) across the three species. The expected log_2_ fold-changes were defined by the experimental design: 0 for human (invariant), 1 for yeast (2-fold decrease in condition β), and −2 for *E. coli* (4-fold increase in condition β). Expected fold-changes were derived from the defined mixture ratios between conditions α and β (30%→15% yeast; 5%→20% *E. coli*), corresponding to log_2_ ratios of 1 and −2, respectively.

We utilized the mean absolute error (MAE) of these fold changes as a global measure of quantification accuracy. Quantification precision was further evaluated using the coefficient of variation (% CV) of precursor intensities across technical replicates, where lower CVs indicate higher reproducibility.

## 3 Results

To systematically evaluate the synergy between instrumentation and software evolution in proteomics, we analyzed a comprehensive benchmark data set spanning eight mass spectrometry platforms, three generations of search engines (X!Tandem, MS-GF+, and Sage), and three postprocessing strategies (no rescoring, Mokapot, and MS^2^ Rescore). This 72-condition experimental matrix (2004–2024) allows us to separate the individual effects of each technology layer across several metrics. All results are available as interactive HTML reports for per-run exploration and as serialized Python objects for programmatic cross-condition analysis on Zenodo (DOI: https://zenodo.org/records/18743931).

### 3.1 Software Advances Deliver Larger Identification Gain on Newer Instrumentation

The most fundamental measure of proteomic performance is the number of confident identifications achieved from a given sample. We assessed identification rates at three hierarchical levels: PSMs, unique peptide sequences, and proteins.

#### 3.1.1 Instrument Advances Drive Broader and Deeper Proteome Coverage

A distinct chronological improvement in identification count is evident across instrument generations (Figure 2; peptide-level data in Figure S1). Comparing instruments using identical software configurations shows substantial gains over time, with the modern instruments (timsTOF Pro, ∼7,000 proteins) identifying more than 3 times as many proteins compared to the earliest platform tested (TripleTOF 5600, ∼2,300 proteins). This establishes instrument innovation as the dominant driver of identification performance. The latest instruments (timsTOF Ultra 2 and Orbitrap Astral) offer high identification rates even with reduced sample loads and short gradient lengths (Table 1).

**Figure 2:**
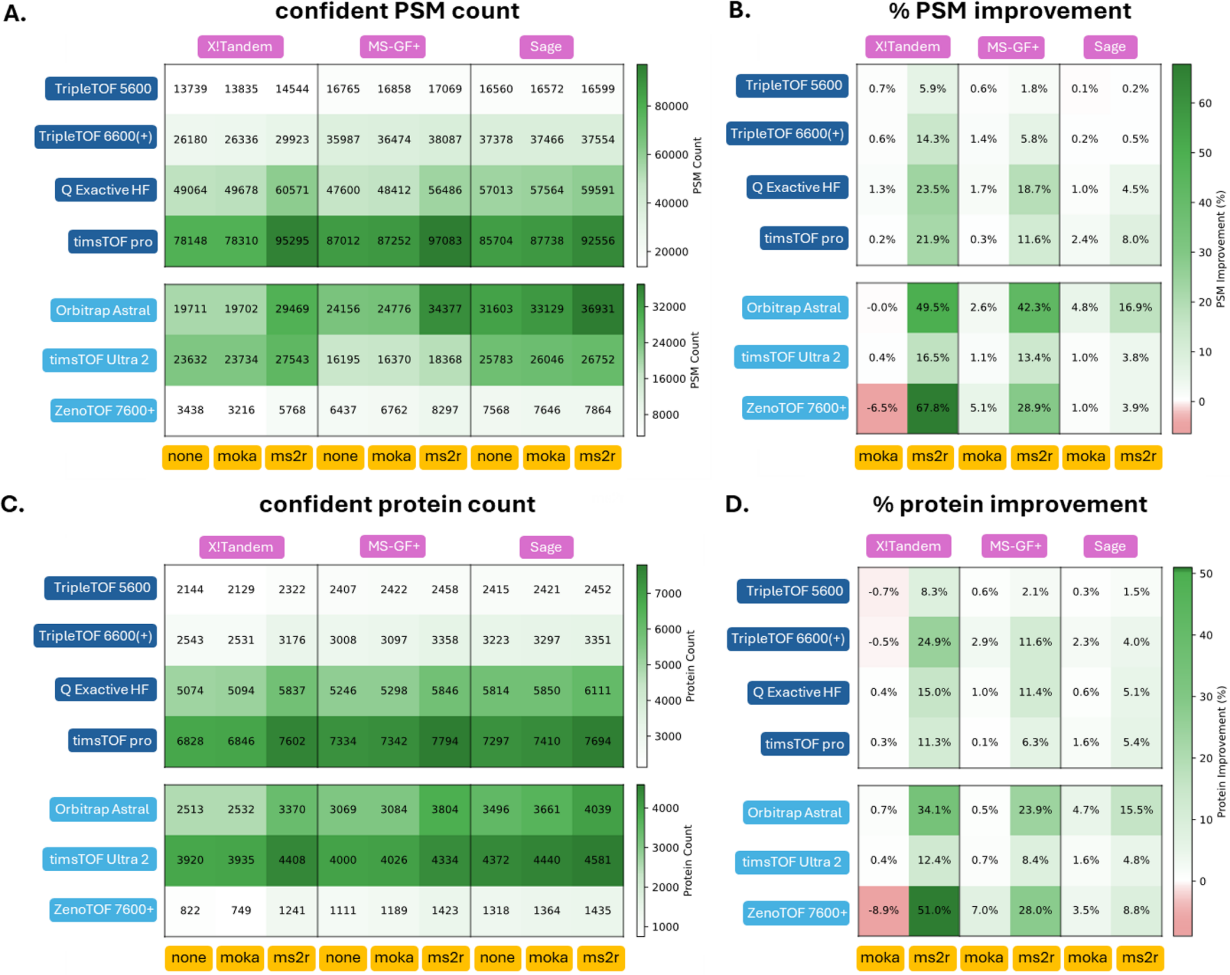
Identification performance across generations of instruments and analysis software. (A, C) Absolute counts of confidently identified PSMs (A) and proteins (C) at 1% FDR (mean of *n* = 6). The figure layout allows for chronological comparison across all axes: (1) instruments (rows) are ordered by commercial release year from oldest (top) to newest (bottom); (2) search engines (major columns) are ordered by release date from left to right (X!Tandem [2004], MS-GF+ [2014], Sage [2023]); and (3) Postprocessing methods (subcolumns) are ordered from baseline to most advanced/recent (none → moka [Mokapot] → ms2r [MS^2^ Rescore]). Color intensity indicates identification count. (B, D) Percentage improvement in identifications relative to the native search engine baseline (no rescoring). Green indicates a gain in identifications, while red indicates a loss. All plots show a visual gap that separates instruments with different acquisition parameters (Table 1).

These identification gains translate into broader and deeper proteome coverage (Figure S2). Proteome-wide sequence coverage rose disproportionately compared to protein counts, increasing approximately 4-fold from the TripleTOF 5600 to the timsTOF Pro (∼1% to ∼4%) versus a 3-fold gain in protein counts. Modern instruments therefore not only identify more proteins but also achieve greater sequence depth per protein. Sequence diversity analysis further indicates that these coverage gains reflect a genuine expansion of the accessible peptide landscape rather than denser sampling of the same sequence space (Supplementary Section 1.1, Figure S3). Coverage uniformity across the identified proteome, however, remains essentially unchanged. In every instrument and software combination, sequence coverage was unevenly distributed: a few proteins were covered almost completely, while most were identified from only one or a few peptides. We measured this imbalance with the Gini coefficient (0 = every protein covered equally, 1 = coverage concentrated in a few proteins), which stayed near 0.45 across all 72 combinations (Δ ≈ 0.05; Supplemental Figure 4). Better instruments and software thus increase how many proteins are found and how much of each is seen, but not how evenly coverage is spread, indicating that this uneven sampling is a fundamental limitation of bottom–up proteomics that current advances do not address.

#### 3.1.2 Modern Search Engines Are More Platform-Robust

When focusing on software effects, we observe that older search engines show more inconsistent performance across instrument platforms, while Sage performs robustly regardless of the platform (Figure 2). X!Tandem performs particularly poorly on SCIEX instruments (TripleTOF 5600, 6600­(+), and ZenoTOF 7600+). MS-GF+ is more consistent than X!Tandem but still underperforms on the timsTOF Ultra 2 data. Both X!Tandem and MS-GF+ struggle under short-gradient, low-sample-load conditions (further examined in Section 4.2). In contrast, Sage consistently matches or exceeds the best results of both older search engines across all platforms, without exhibiting performance collapse on any platform. Sage also processes data sets substantially faster than both older search engines (Figure S5).

#### 3.1.3 Rescoring Gains Are Larger on Newer Instruments

The magnitude of rescoring improvements follows a diagonal gradient across the improvement matrices, from the greatest gains in the lower-left corner (older search engines on newer instruments) to the smallest gains in the upper-right corner (modern search engines on older instruments). This reveals two complementary trends: first, older search engines benefit more dramatically from rescoring than modern algorithms such as Sage, which natively already incorporate simple data-driven rescoring; second, regardless of search engine sophistication, rescoring delivers greater improvements on modern instruments than on older platforms. Additionally, high-throughput acquisition conditions benefited considerably from rescoring. Together, these observations suggest that instrument and software advances provide benefits synergistically, rather than substitutively.

### 3.2 Rescoring is Most Impactful for Low-Abundance Precursors and for High-Throughput Acquisition Conditions

Beyond counting total identifications, a critical question is whether rescoring improves detection uniformly across the precursor intensity range or preferentially enhances identification of specific subpopulations. Figure 3 and Figure S6 clearly show that rescoring predominantly improves detection of low-abundance precursors. The percentage improvement in PSM counts increases from high- to low-intensity bins, a pattern that is consistent across all search engines and instrument platforms.

**Figure 3:**
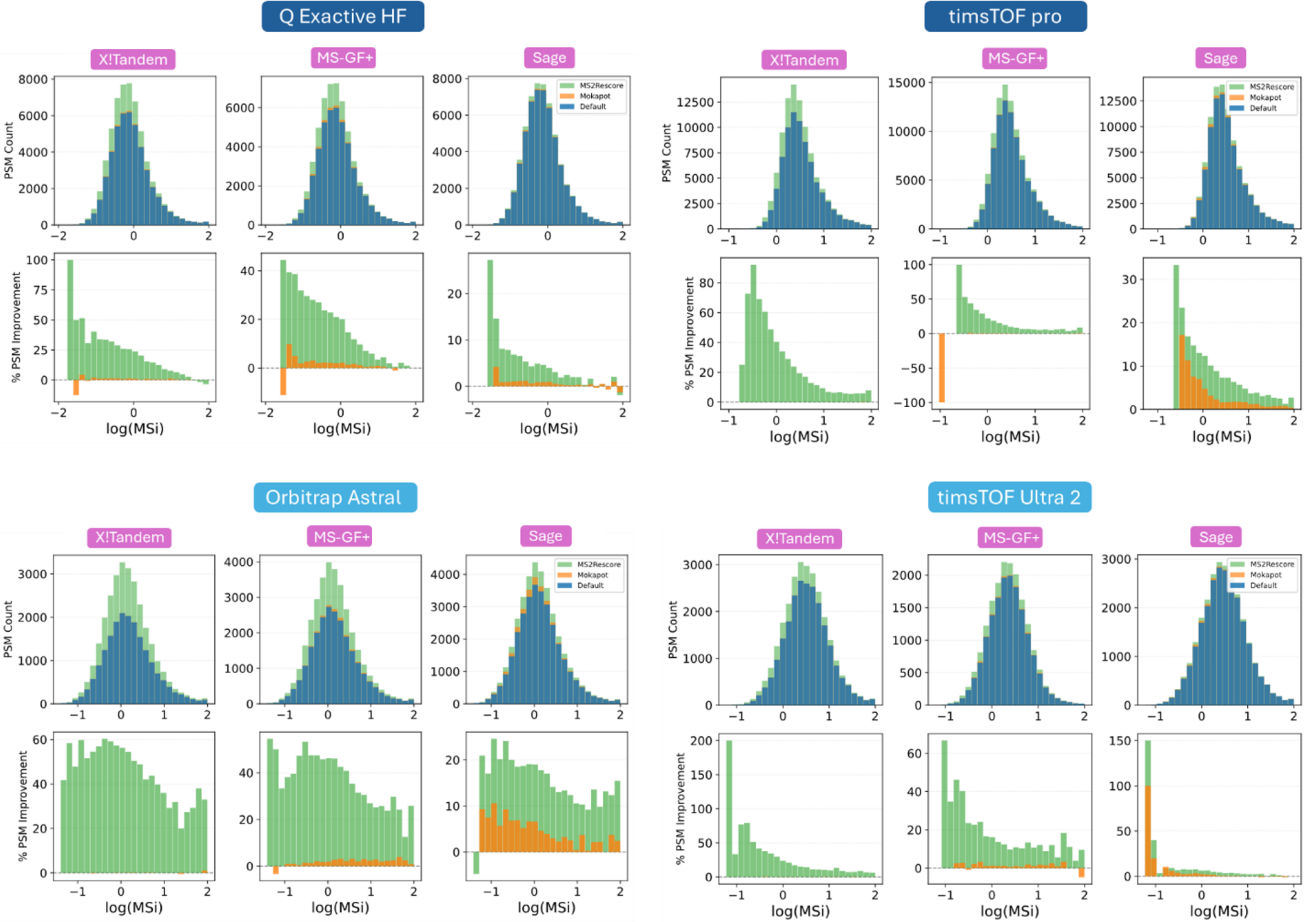
ML-based rescoring preferentially recovers low-abundance precursors. Representative examples from four instrument platforms showing distributions of log-transformed normalized precursor intensity (log­(MSi)) for baseline (blue), Mokapot (orange), and MS^2^ Rescore (green) conditions (upper panels) and bin-wise percentage improvement in PSM counts relative to baseline (lower panels). Data shown are from the first replicate of condition α for each platform. Results are shown for three search engines (X!Tandem, MS-GF+, Sage).

This intensity-dependent benefit suggests that rescoring should provide its largest gains under acquisition conditions that shift the precursor population toward lower abundances, such as the short gradients and minimal sample loads increasingly used in high-throughput proteomics workflows. To test this, we compared identification performance across four acquisition conditions varying in sample load (50 ng vs 250 pg) and gradient length (5 min vs 15 min) on the Orbitrap Astral and timsTOF Ultra/Ultra 2 (Figure S7). Here, rescoring benefits generally increased as the sample load decreased. One notable exception was observed at the 250 pg sample load, where rescoring after Sage provided almost no additional benefit.

### 3.3 Identification Gains from Rescoring Extend into Intensity Ranges Where Reliable Quantification is Challenging

To evaluate how innovations across each layer impact quantification, we performed label-free quantification with FlashLFQ,^27^ comparing observed log_2_ fold-changes (log_2_FC) to expected values to assess accuracy across all instrument–software combinations.

Gains in peptide identification were generally accompanied by a decrease in quantification accuracy, as observed through increased mean absolute errors in the log_2_FC estimation (Figure 4; Figures S8 and S9). This trend was most pronounced on platforms where rescoring delivered substantial gains in sensitivity, while platforms where rescoring had minimal impact on identification counts (TripleTOF 5600, 6600 (+)) showed little change in quantification error. Stratification of peptides by intensity (Figure 4B) clarifies the mechanism behind this trade-off. Corroborating our findings in Section 4.2, rescoring algorithms excel at recovering low-abundance species. By bringing these precursors above the FDR threshold, rescoring allows them to be quantified. Crucially, these low-abundance peptides exhibit substantially higher quantification uncertainty, with both mean absolute error and coefficient of variation increasing several-fold at the lowest precursor intensities across all conditions (Figure 4B and Figure S9). The population-level increase in quantification error therefore reflects the compositional shift toward inherently noisier measurements rather than a degradation of the quantification quality for commonly identified species.

**Figure 4:**
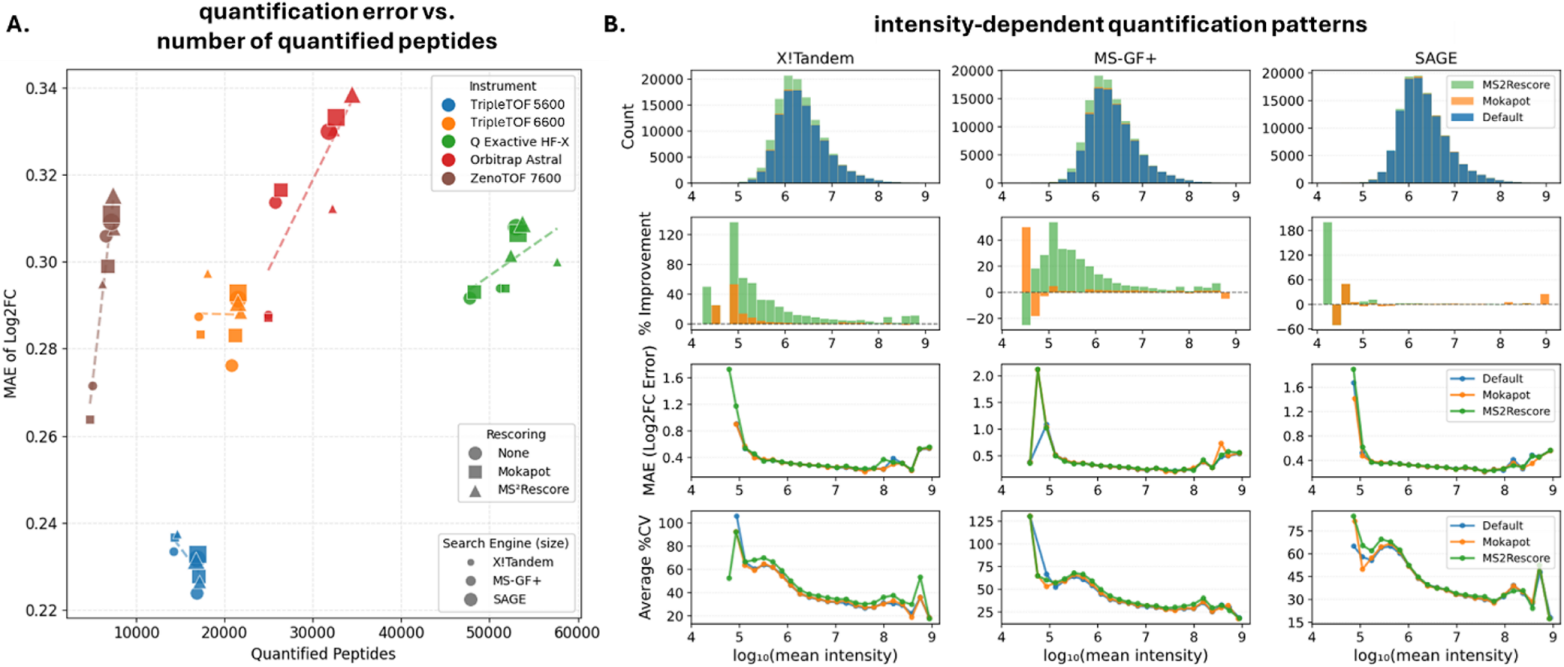
Trade-off between identification depth and quantification accuracy. (A) Scatter plot showing the trade-off between the number of quantified peptides (*x*-axis) and quantification accuracy (MAE of log_2_ fold-change, *y*-axis) across all instrument–software combinations. Marker color indicates the instrument platform, marker shape indicates the rescoring condition, and marker size indicates the search engine. Dashed lines show linear regressions fitted per instrument, illustrating how software improvements increase peptide counts but at the cost of higher quantification error. (B) Intensity-dependent quantification patterns on the Q Exactive HF-X across the three search engines (columns). From top to bottom: distribution of quantified peptides per precursor intensity bin (averaged across replicates), percentage improvement in peptide counts after rescoring relative to the baseline (no rescoring), MAE of log_2_ fold-change as a function of precursor intensity, and average coefficient of variation (%CV) across technical replicates as a function of precursor intensity.

## 4 Discussion

The comprehensive evaluation across eight mass spectrometry platforms, three generations of search engines, and multiple rescoring strategies reveals a central finding: instrument and software advances in proteomics produce synergistic rather than substitutive benefits. Modern instruments do not render sophisticated algorithms obsolete, nor do advanced computational methods fully compensate for older instruments. Instead, each layer of technological innovation creates new opportunities and challenges that subsequent layers can exploit or address.

This synergy manifests across multiple performance measurements—identification rates, sensitivity, and coverage depth—but several questions remain open, such as why modern instruments benefit more from ML-based rescoring than older platforms, why Sage maintains robust performance across all instrument types while X!Tandem and MS-GF+ show platform-specific degradation, and why rescoring improvements concentrate in the low-abundance precursor regime. In the sections below, we formulate various hypotheses to address these questions.

### 4.1 Data-Driven Scoring Explains the Shift from Platform-Specific to Universal Search Engine Performance

The contrasting performance of X!Tandem, MS-GF+, and Sage across different instrument platforms (Section 4.1) likely reflects fundamental differences in scoring algorithm adaptability. X!Tandem’s static scoring function applies identical heuristic rules across all instruments, performing well only when spectrum properties align with its fixed assumptions about fragmentation patterns. This lack of adaptability explains the broad variability observed across platforms. Additionally, X!Tandem’s much lower performance on SCIEX instruments can likely be explained by a specific technical incompatibility: MSConvert’s missing encoding of precursor charge states during.wiff to mzML conversion forces the search engine to test multiple charge hypotheses per spectrum, effectively expanding the search space.

MS-GF+ improves on this rigidity but remains constrained by its reliance on precomputed statistical tables of fragment ion intensity distributions derived from large amounts of data. These tables encode platform-specific spectrum characteristics; when applied to instruments with different fragmentation physics or detector properties, the distributions may poorly model observed spectra, thereby degrading performance. MS-GF+ thus remains sensitive to the match between the platforms and data used to generate its statistical tables and the target instruments being analyzed.

Sage’s universal robust performance across all platforms can be attributed to its data-driven scoring approach. Unlike X!Tandem’s fixed heuristics or MS-GF+‘s collected statistical tables, Sage employs linear discriminant analysis to dynamically derive a scoring function optimized for each data set. By learning feature weights directly from the data rather than relying on predefined assumptions, Sage automatically accommodates platform- and run-specific spectrum characteristics without requiring instrument-specific optimization.

### 4.2 Proteome Coverage Improves but Uniform Sampling Remains a Fundamental Limitation

While protein identification has improved substantially over the past decade, complete proteome coverage remains elusive (Figure S2). Our proteome-wide sequence coverage analysis reveals that even modern instruments map only about 4% of the combined reference proteome’s sequence, compared to roughly 1% on 2010-era quadrupole time-of-flight platforms. Though this reflects meaningful progress, it underscores the considerable gap between current capabilities and comprehensive proteome characterization, particularly when also considering post-translational modifications.

Equally informative is the coverage uniformity analysis (Figure S4): Gini coefficients demonstrate minimal improvement across instrument generations and virtually no change with software enhancements. This persistent inequality, independent of technological advances, indicates that improved instrumentation and algorithms expand the breadth of proteome sampling without fundamentally altering the underlying biases. Achieving more uniform proteome coverage will likely require combining orthogonal strategies, such as combining multiple proteases, fragmentation techniques, and middle-down and top-down approaches.^29^

### 4.3 The Noisy Spectrum Hypothesis Explains Rescoring Trends—with One Exception

The results from Sections 3.2 and 3.3 revealed that rescoring gains concentrate consistently in the low precursor intensity range, with benefits diminishing toward high-abundance peptides. A straightforward explanation is that low-abundance precursors produce noisy spectra, in which background signal and coeluting interference add irrelevant peaks that obscure the true fragments, so that standard scores based on peak counts and matched intensities lose discriminative power. Here, ML-based rescoring can provide additional discriminative power through orthogonal evidence. This “noisy spectrum” hypothesis successfully explains several observations: (i) the exponential decay of rescoring benefits from low to high intensities (Figures 3 and 4), and (ii) the substantial rescoring gains observed for 50 ng samples on modern instruments compared to higher sample loads (Figure 2 and Figure S7).

However, this explanation proves to be incomplete under extreme conditions. The 250 pg sample load experiments (Section 4.2) reveal that additional rescoring provides minimal improvement over Sage, despite these conditions producing predominantly low-quality spectra. One likely explanation is that the benefit comes mainly from rescoring and retention time information (already performed by Sage natively), while fragment intensity prediction does not contribute. At such low intensities, the recorded fragment intensities may no longer match the predicted patterns, removing the added value this evidence provides at higher abundances. Further research on intensity patterns under extremely low-input conditions would be required to confirm these hypotheses.

### 4.4 Rescoring Extends Identification Depth Beyond Current Quantification Limits

The inverse relationship between identification depth and quantification accuracy (Section 4.3) represents an underappreciated consequence of pushing detection boundaries with sophisticated software methods. Our intensity-stratified analysis (Figure 4B) explains the underlying mechanism: rescoring methods do not reduce the accuracy of commonly identified peptides but rather expand the quantified population by recovering low-abundance precursors that exhibit 2–3-fold higher quantification errors and coefficients of variation. The population-level increase in error stems entirely from this compositional change.

This has a critical implication: mean quantification errors are not representative of data set-wide algorithmic performance, as error metrics are highly intensity-dependent. A data set quantifying predominantly high-abundance peptides may show low mean error despite poor algorithmic performance, while one extending into the low-abundance regime may show elevated mean error despite excellent performance. This nuance should be considered when interpreting the label-free quantification results.

Notably, this trade-off is not specific to FlashLFQ: the ProteoBench^28^ DDA quantification module, which benchmarks multiple quantification tools on the same data set, shows a consistent relationship between identification depth and quantification accuracy (https://proteobench.cubimed.rub.de/Quant_LFQ_DDA_ion_QExactive).

### 4.5 Instrument–Software Synergy May Extend Beyond Data-Dependent Acquisition

While our evaluation focused exclusively on DDA workflows, we hypothesize that the observed patterns of instrument–software interaction extend to data-independent acquisition (DIA) methods. DIA fragments all precursors within defined mass windows rather than selectively targeting abundant ions, eliminating stochastic sampling and inherently capturing more low-abundance peptides and greater spectrum heterogeneity, conditions under which ML-based rescoring provides stronger gains. Additionally, DIA spectra contain fragment ions from multiple coisolated precursors, creating highly chimeric spectra where discriminative scoring becomes even more critical. These characteristics suggest that the synergistic relationship between instrumentation and software we documented for DDA may manifest even more strongly in DIA workflows. Notably, ML-based scoring is already standard practice in widely adopted DIA pipelines such as Spectronaut,^30^ DIA-NN,^31^ and AlphaDIA.^32^

## 5 Conclusions

This systematic evaluation across eight mass spectrometry platforms, three search engine generations, and multiple rescoring strategies reveals that advances in mass spectrometry-based proteomics instruments and software compound rather than substitute for one another. Each technological layer amplifies the capabilities of the others, producing compounded gains in sensitivity and coverage.

ML-based rescoring methods deliver the most pronounced benefits on modern platforms, where increased sensitivity and scan speed detect a larger population of low-abundance precursors that produce noisier, more challenging spectra—precisely the regime where feature-based discrimination provides the greatest advantage. However, this expanded detection depth introduces a trade-off between coverage and quantification accuracy, as inclusion of low-intensity peptides inherently increases the average quantification error. Meanwhile, comprehensive and balanced proteome coverage remains elusive: while newer instruments and algorithms broaden the detectable proteome, sequence coverage across proteins remains uneven, highlighting biases that incremental innovations alone cannot resolve. Future progress in proteomics will depend on coordinated coevolution between instrumentation and software, combining advanced acquisition strategies with adaptive scoring algorithms capable of leveraging the expanding complexity of modern LC-MS/MS data.

## Supplementary Material



## Notes

Supplementary Methods 1.1. Sequence diversity analysis. Supplementary Table 1.: chromatographic configurations for DDA acquisition across LC-MS/MS platforms. Supplementary Table 2: docker image tags used in the Nextflow pipeline. Supplementary Table 3: software tools and versions used in this study. Supplementary Table 4: MSConvert parameters for converting vendor-specific mass spectrometry file formats to open formats. Supplementary Table 5: rescoring features used with Mokapot. Figure S1: peptide identification performance across mass spectrometry platforms, search engines, and rescoring methods. Figure S2: proteome coverage metrics across instrument and software configurations. Figure S3: sequence diversity metrics across instrument and software configurations. Figure S4: coverage uniformity across the identified proteome (Gini coefficients). Figure S5: computational performance comparison of peptide search engines. Figure S6: machine learning-based rescoring preferentially recovers low-abundant precursors. Figure S7: impact of sample load and gradient length on identification performance and rescoring effectiveness across modern instruments. Figure S8: trade-off between identification depth and quantification accuracy. Figure S9: intensity-dependent quantification patterns across mass spectrometry platforms. Figure S10: validation of sparse similarity matrix approximation for sequence diversity metrics (PDF)
