# Supplementary material for: Instrument–Software Synergy in Proteomics: Systematic Evaluation across Mass Spectrometry Platforms, Search Engines, and Rescoring Methods
Source: J Proteome Res. 2026 Jul 14;25(8):4251–61. doi: 10.1021/acs.jproteome.6c00295 (PMC13459558; doi:10.1021/acs.jproteome.6c00295)
Supplement: Supplementary file 1 [file pr6c00295_si_001.pdf]

Supplementary: Instrument-Software Synergy in Proteomics: Systematic Evaluation Across Mass Spectrometry Platforms, Search Engines, and Rescoring Methods

Sander Heyndrickx[1,2], Robbin Bouwmeester[1,2], Arthur Declercq[1,2], Robbe Devreese[1,2], Magnus Palmblad[3], Wout Bittremieux[4], Tess AV Afanasyeva[5], Joel Lapin[7], Arzu Tugce Guler[8,9,10], Pierre-Olivier Schmit[11], Christine Carapito[12,13], Lennart Martens*[1,2,12,13], and Ralf Gabriels[1,2]

[1] CompOmics, VIB-UGent Center for Medical Biotechnology, VIB, 9000 Ghent, Belgium
[2] Department of Biomolecular Medicine, Faculty of Medicine and Health Sciences, Ghent University, 9000 Ghent, Belgium
[3] Center for Proteomics and Metabolomics, Leiden University Medical Center, 2311 ZA Leiden, Netherlands
[4] Department of Computer Science, University of Antwerp, 2020 Antwerpen, Belgium
[5] Sanquin Research and Landsteiner Laboratory, Amsterdam UMC, University of Amsterdam, 1066 CX Amsterdam, Netherlands
[7] Computational Mass Spectrometry, TUM School of Life Sciences, Technical University of Munich, 85354 Freising, Germany
[8] Institute for Experiential AI, Northeastern University, Boston, Massachusetts 02115, United States
[9] Department of Pathology, Boston Children's Hospital, Boston, Massachusetts 02115, United States
[10] Harvard Medical School, Boston, Massachusetts, 02115, United States
[11] Bruker France SAS, 67160 Wissembourg, France
[12] BioOrganic Mass Spectrometry Laboratory (LSMBO), IPHC UMR 7178, University of Strasbourg, CNRS, 67000 Strasbourg, France
[13] Infrastructure Nationale de Protéomique ProFI − UAR 2048, 67087 Strasbourg, France

* To whom correspondence should be addressed:
Tel: +32 9 264 93 58
Email: lennart.martens@vib-ugent.be
Address: Technologiepark 75, 9052 Ghent, Belgium

ORCIDs
Sander Heyndrickx: 0009-0004-1818-8619
Robbin Bouwmeester: 0000-0001-6807-7029
Arthur Declercq: 0000-0002-9376-1399
Robbe Devreese: 0000-0002-3432-1502
Magnus Palmblad: 0000-0002-5865-8994
Wout Bittremieux: 0000-0002-3105-1359
Tess Afanasyeva: 0000-0003-3962-0779
Joel Lapin: 0009-0009-5013-238X
Arzu Tugce Guler: 0000-0002-6043-7824
Pierre-Olivier Schmit: 0000-0003-2269-2389
Christine Carapito: 0000-0002-0079-319X
Lennart Martens: 0000-0003-4277-658X
Ralf Gabriels: 0000-0002-1679-1711

Table of content

Supplementary information 3
1 Supplementary Methods 3
1.1 Sequence Diversity 3
2 Supplementary figures 4
3 Reference 11

Supplementary information

1 Supplementary Methods

1.1 Sequence Diversity

Beyond the coverage analyses presented in the main text, an additional question is whether technological advances recover more diverse peptide sequences or re-sample similar ones. To address this, we quantified pairwise sequence similarity across all identified peptides and derived two complementary diversity metrics.

Similarity computation. Computing all-against-all pairwise similarities becomes infeasible for large peptide sets. Since both diversity metrics depend on identifying the most similar sequences in the dataset, random subsampling of pairs would likely miss these nearest neighbors. To address this, we used a two-step approach to construct a sparse similarity matrix that efficiently identifies each peptide's nearest neighbors.

In the first step, candidate pairs of similar peptides were identified using an approximate string-matching approach adapted from text similarity analysis. Here, each peptide sequence was represented as a vector of overlapping three-amino-acid subsequences weighted by term frequency-inverse document frequency (TF-IDF) as implemented in scikit-learn's TfidfVectorizer[1] (v1.7.2). Cosine similarity between these vectors was then used to retain only the top 5% most similar peptides per sequence.

In the second step, the normalized Levenshtein edit distance was computed for each candidate pair, assigning equal weight to insertions, deletions, and substitutions, and normalizing by the length of the longer peptide in the pair. Edit distances were then converted to similarity scores as $S = 1 - d$, where d is the normalized edit distance, yielding values ranging from 0 (completely different) to 1 (identical). The resulting sparse similarity matrix was validated against dense similarity matrices computed on dataset subsets (Supplemental Figure 10).

Local sequence diversity was quantified as the mean nearest-neighbor similarity. For each peptide, we identified its most similar counterpart from the sparse similarity matrix described above. The average of these maximum similarities across all peptides serves as a measure of local redundancy, where lower values indicate more diverse peptide populations.

Global sequence diversity was quantified using a sequence-thresholded clustering approach. Using the sparse similarity matrix described above, we constructed networks where peptides are nodes and edges connect pairs exceeding a given similarity threshold. The number of disconnected clusters was counted at thresholds of 40%, 50%, 60%, 70%, 80%, and 90%, and normalized by the total number of nodes in the network. The area under the curve (AUC) of these normalized cluster counts across thresholds summarizes global diversity: higher AUC values indicate that the network fragments rapidly with increasing thresholds, reflecting high diversity, while lower values indicate more homogeneous sequence composition.

A limitation of these sequence similarity metrics is that they treat all amino acid substitutions equivalently and do not account for the physicochemical properties of the residues.

Nevertheless, the Levenshtein-based approach provides an interpretable first-order measure of sequence dissimilarity. Results are shown in Supplemental Figure 3.

2 Supplementary figures

Supplemental Table 1. Chromatographic configurations for DDA acquisition across different LC-MS/MS platforms. Summary of liquid chromatography setups used for data-dependent acquisition (DDA) in this study.

instrument	sample load	LC system	column	flow rate	mobile phase A	mobile phase B	gradient
**SCIEX TripleTOF 5600**	5 µg	Eksigent NanoLC 400	YMC TriArt C18 (id 300 µm, length 15 cm, 3 µm); trap: YMC TriArt C18 guard (id 500 µm, length 5 mm, 3 µm)	5 µL/min	0.1% FA + 3% DMSO in water	0.1% FA in ACN	2–30% B in 120 min
**SCIEX TripleTOF 6600+**	5 µg	Eksigent NanoLC 425	Phenomenex Luna Omega Polar C18 (150 × 0.3 mm, 3 µm)	5 µL/min	0.1% FA in water	0.1% FA in ACN	2–30% B in 120 min
**Thermo Orbitrap QE HF-X**	1 µg	UltiMate 3000 (NCS-3500RS)	Acclaim PepMap C18 (id 75 µm, length 50 cm, 2 µm); trap: (id 300 µm × 5 mm)	350 nL/min	0.1% FA in water	0.1% FA in ACN	2–30% B in 120 min
**Bruker timsTOF Pro**	1 µg	Acquity UPLC M-Class	nanoEase M/Z HSS C18 T3 (100 Å, 1.8 µm, 75 µm × 250 mm); trap: nanoEase M/Z Symmetry C18 (100 Å, 5 µm, 180 µm × 20 mm)	400 nL/min	0.1% FA in water	80% ACN + 0.1% FA	2–30% B in 120 min
**Thermo Orbitrap Astral**	50 ng	Vanquish Neo	50 cm µPAC™ column (180 µm bed width)	250 nL/min	0.1% FA in water	0.1% FA in 80% ACN	4–40% B in 5 or 15 min
**Thermo Orbitrap Astral**	250 pg	Vanquish Neo UHPLC	50 cm µPAC™ column (180 µm bed width); trap: µPAC™ Trapping Column	200 nL/min	0.1% FA in water	0.1% FA in 80% ACN	3.7–37.5% B in 5 or 15 min
**Bruker timsTOF Ultra**	250 pg	nanoElute 2	5 cm Aurora Rapid CSI column (IonOpticks; 75 µm id, 1.7 µm, 120 Å)	250 nL/min	0.1% FA in water	0.1% FA in ACN	3–30% B in 5 or 15 min
**Bruker timsTOF Ultra-2**	50 ng	nanoElute	5 cm Aurora Rapid column (IonOpticks; 150 µm id, 1.7 µm, 120 Å)	200 nL/min	0.1% FA in water	0.1% FA in ACN	3–30% B in 5 or 15 min
**SCIEX ZenoTOF 7600+**	50 ng	Acquity M-Class UPLC	Kinetex XB-C18 (15 cm × 300 µm, 2.6 µm; Phenomenex)	5 µL/min	0.1% FA in water	0.1% FA in ACN	3–30% B in 5 or 15 min

Supplemental Table 2: Overview of the Docker image tags used in the Nextflow pipeline.

Tool	Tag	Image	SHA256
SearchGUI	4.3.15--hdfd78af_0	quay.io/biocontainers/searchgui	sha256:55fcb7b79922725737ddde120305f85021feb1cf30afc38612fde4233d 151b1b
MSConvert	proteowizard/pwiz-skyline-i-agree-to-the-vendor-licenses	skyline_25.1.0.224-519d29b	sha256:d1ffa4b6a0dad2fd578ae22149e64e0f1fa60920eef4b0014fb4fffc27f3cc88
FlashLFQ	smithchemwisc/flashlfq	2.1.4	sha256:8dff05efc205bd62094d51aced17f57c377f04eeff231ca00e78a21e04f40b87

Supplemental Table 3. Software tools and versions used in this study. Versions correspond to the releases executed in the analysis pipeline (as of October 2025).

Tool	Initial release	Latest release at time of analysis (Oct 2025)	Version used
X!Tandem	2003 (first release 2003.05.01)	ALANINE (2017.02.01)	VENGEANCE (2015.12.15)
MS-GF+	2014[1] (GitHub releases from v2017.01.10)	v2024.03.26	v2024.03.26 (= latest)
Sage	v0.3.1 (2022-10-07, first GitHub release)	v0.14.7 (2024-03-15)[2]	v0.14.6 (2024-02-03)
SearchGUI / SearchCLI	2011[3] (v1.1, 2010-08-23)	4.3.15 (2025-05-14)	4.3.15 (= latest)
MSConvert (ProteoWizard)	2008	ProteoWizard 3.0[4] (continuous; 2025 build)	ProteoWizard 3.0 (pwiz-skyline image skyline_25.1.0.224-519d29b)
Mokapot	2020-07-08 (0.1a0)	0.10.0 (2023-09-11)	0.10.0 (= latest)
MS²Rescore	2021-01-26 (2.0.0b2)	3.2.0.post1 (2025-10-22)	3.1.5
MS²PIP	2013[5] (first PyPI 3.0.6, 2019-12-29)	4.1.0 (2025-01-20)	4.1.0 (= latest)
DeepLC	2019-12-15 (0.1.1-dev1)	3.1.13 (2025-09-01)	3.0.8
IM2Deep	2024-02-01 (0.1.0)	1.1.0 (2025-08-08)	0.3.1
FlashLFQ	2017-08-10 (0.1.16)	2.1.4 (2025-07-25)	2.1.4[6]
scikit-learn	2011-09-22 (0.9)	1.7.2 (2025-09-09)	1.7.2 (= latest)

Supplemental Table 4. MSConvert parameters for converting vendor-specific mass spectrometry file formats to open formats. Parameters include compression settings, peak picking filters, and format-specific options for ion mobility data processing and charge state prediction.

vendor format	open format	CLI conversion parameters
.raw	.mzML	--mzML --32 --zlib --filter "peakPicking true 1-"
.d	.mzML	--mzML \ --32 \ --zlib \ --combineIonMobilitySpectra \ --filter "scanSumming precursorTol=0.05 scanTimeTol=10" \ --filter "peakPicking true 1-" \
.wiff/.wiff.scan	.mzML	--mzML --32 --zlib --filter "peakPicking true 1-"
.wiff/.wiff.scan	.mgf	--mgf \ --32 \ --zlib \ --filter "peakPicking vendor msLevel=1-" \ --filter "chargeStatePredictor overrideExistingCharge=false maxMultipleCharge=4 minMultipleCharge=2 singleChargeFractionTIC=0.9" \ --filter "titleMaker <RunId>.<ScanNumber>.<ScanNumber>.<ChargeState> File:\"<SourcePath>\", NativeID:\"<Id>\"" \

Supplemental Table 5. Rescoring features used with Mokapot. For each search engine, the listed features were included alongside native engine scores during semi-supervised rescoring with Mokapot. charge_n denotes

the numeric precursor charge state; charge_2 through charge_5 are one-hot encoded indicator variables for each charge state; abs_ms1_error_ppsm is the absolute precursor mass error in parts per million. For Sage, all native Sage rescoring features were additionally included. Note that database searches were configured with a maximum precursor charge of 4; charge_5 is included as a feature because Mokapot's basic feature generator encodes it by default, though it is effectively unused.

search engine	(mokapot rescoring features)
X!Tandem	charge_n, charge_2, charge_3, charge_4, charge_5, abs_ms1_error_ppsm, search_engine_score
MS-GF+	charge_n, charge_2, charge_3, charge_4, charge_5, abs_ms1_error_ppsm, search_engine_score
Sage	sage features, charge_n, charge_2, charge_3, charge_4, charge_5, abs_ms1_error_ppsm, search_engine_score

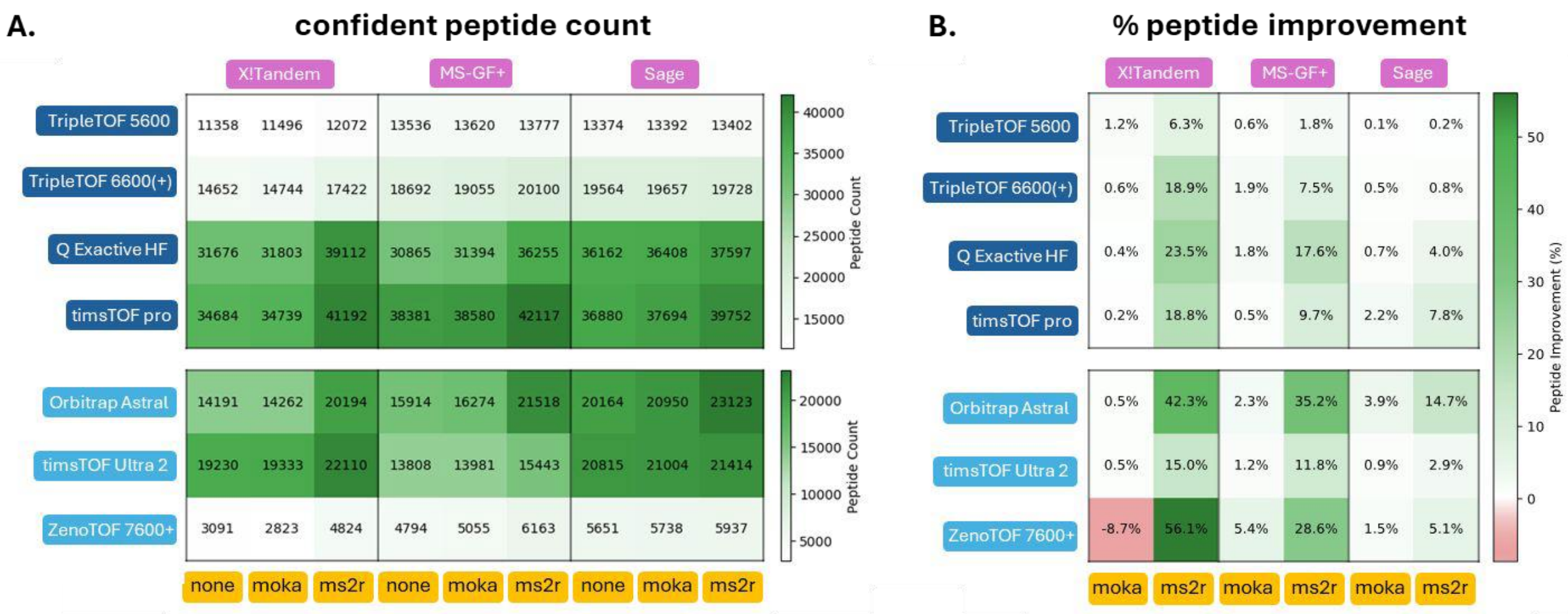

Supplemental Figure 1. Peptide identification performance across mass spectrometry platforms, search engines, and rescoring methods. Absolute counts (left panels) and percentage improvement relative to baseline (right panels), averaged across six replicates. Results are shown for three search engines (X!Tandem, MS-GF+, Sage) and three post-processing approaches (none, moka=Mokapot, ms2r=MS²Rescore abbreviated as ms2r). A visual gap separates instruments with different acquisition parameters: upper instruments used higher sample loads and 120-minute gradients, while lower instruments used 50 ng loads and 15-minute gradients.

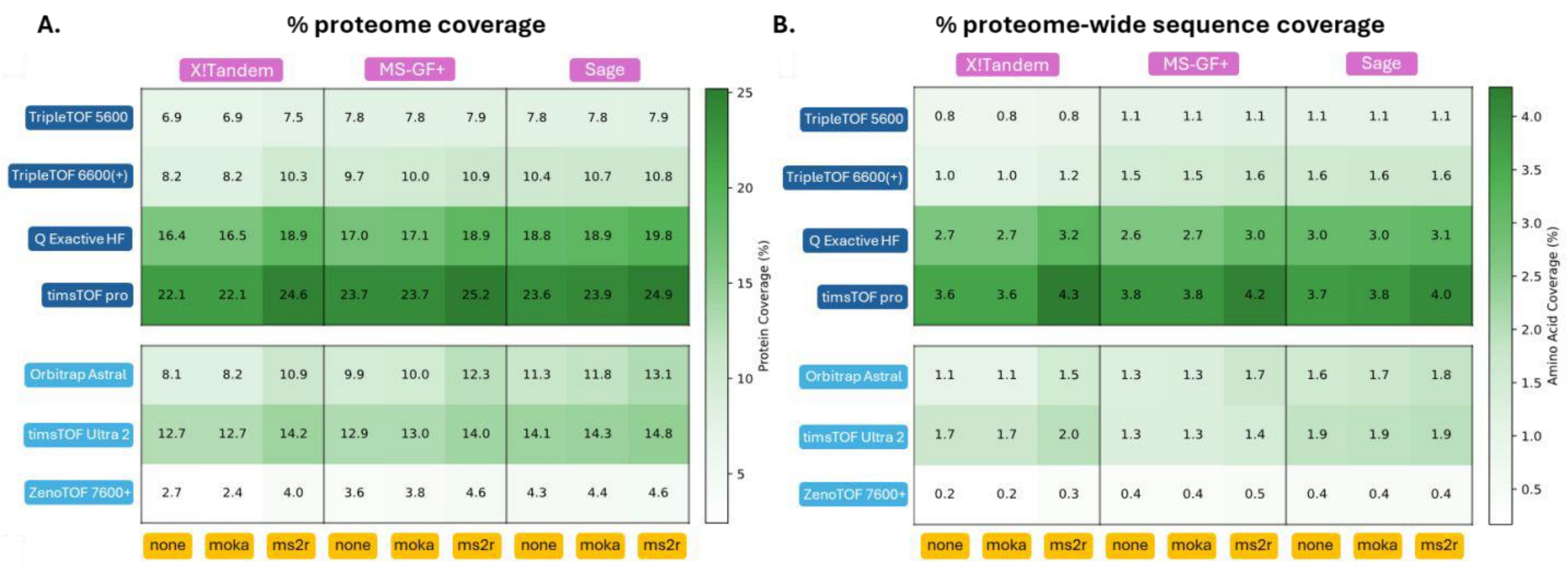

Supplemental Figure 2. Percentage Proteome coverage metrics across instrument and software configurations. (A) Proteome coverage showing the percentage of combined reference proteins (human, yeast, and *E. coli*) confidently identified at 1% FDR (mean n=6). **(B)** Proteome-wide sequence coverage representing the percentage of total reference proteome residues covered by at least one confidently identified peptide, averaged across six replicates. For both panels, color intensity indicates coverage depth. **All plots** show a visual gap that

separates instruments with different acquisition parameters (Table 1). Additionally, the layout of instruments (rows) and software configurations (columns) is identical to Figure 2 (chronological ordering).

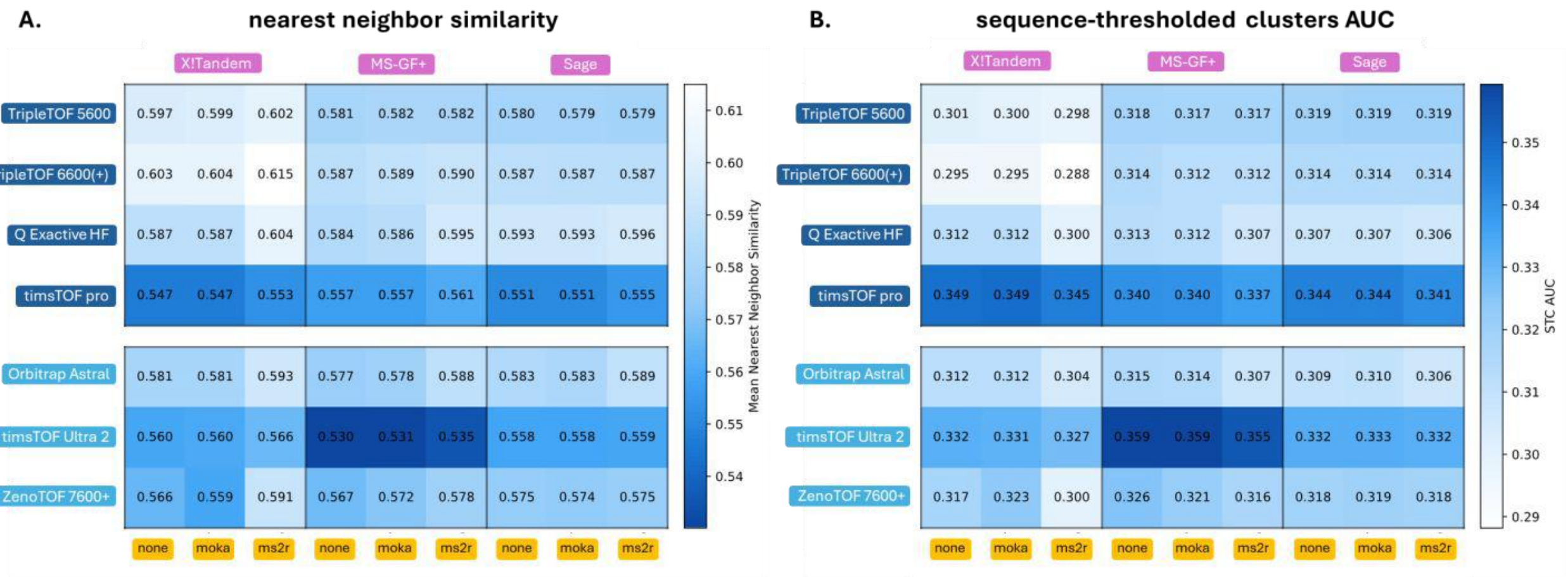

Supplemental Figure 3. Sequence diversity metrics across instrument and software configurations. (A) Mean nearest neighbor similarity showing local sequence relationships, (mean of n=6). Lower values (darker blue) indicate that identified peptides have further sequence neighbors. **(B)** Sequence-thresholded cluster area under the curve quantifying global sequence diversity, (mean of n=6). **Both plots** show a visual gap that separates instruments with different acquisition parameters (Table 1). Higher values (darker blue) indicate greater overall diversity, with peptides rapidly disconnecting into isolated clusters as similarity thresholds increase. Instruments are ordered chronologically (top to bottom), with results shown for three search engines and three post-processing approaches within each platform.

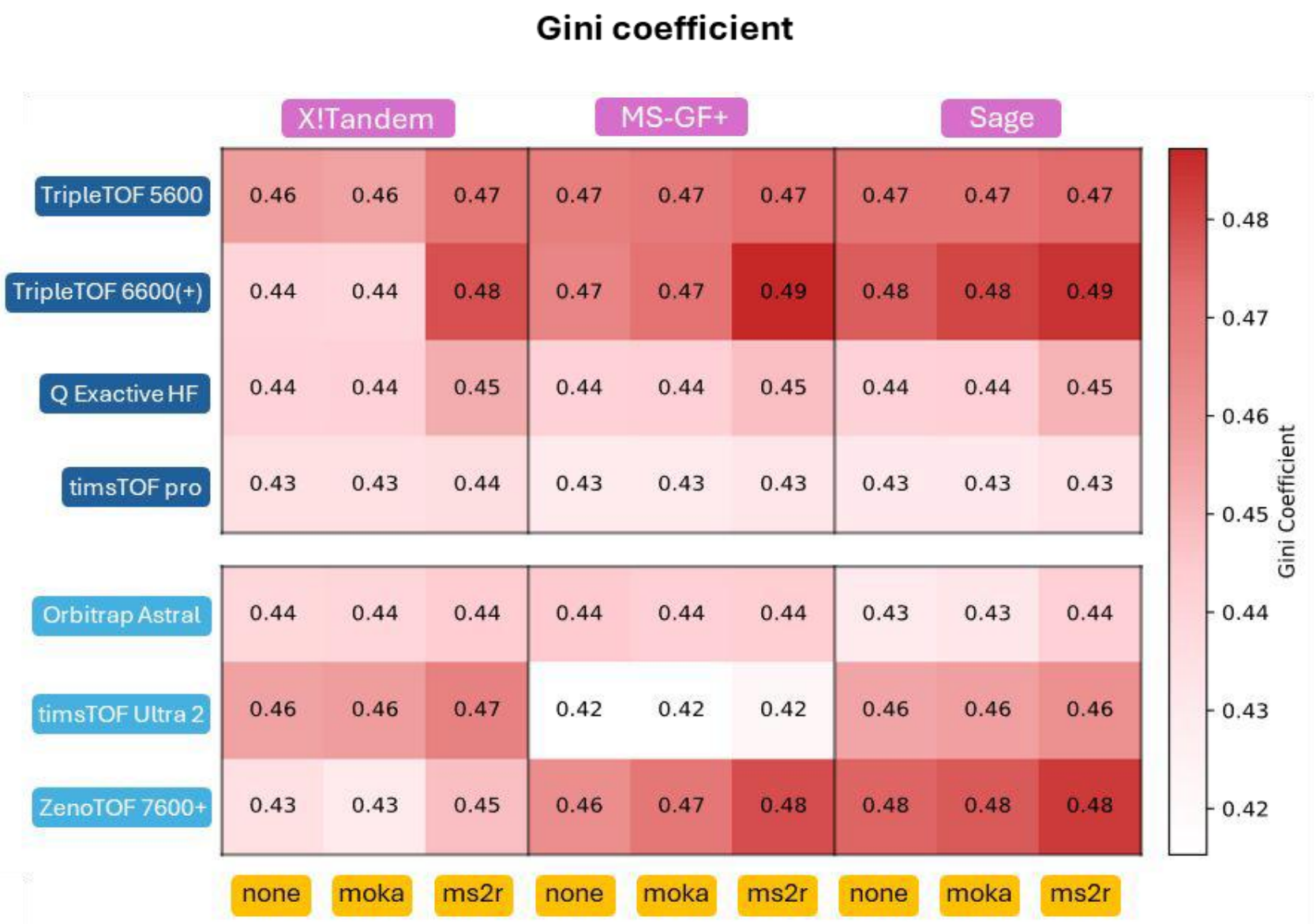

Supplemental Figure 4. Coverage uniformity across the identified proteome. Gini coefficients quantify the distribution of amino acid coverage across confidently identified proteins (1% FDR), averaged across six replicates. Lower values (lighter shading) indicate more uniform sequence coverage across the proteome, while higher values (darker shading) reflect greater inequality with coverage concentrated in a subset of highly sampled proteins. Instruments are ordered chronologically (top to bottom), with results shown for three search engines (X!Tandem, MS-GF+, Sage) and three post-processing approaches (none, moka=mokapot, ms2r=MS²Rescore) within each platform. A visual gap separates instruments with different acquisition parameters (Table 1), precluding direct performance comparison between upper (1-5 µL, 120-min) and lower instruments (50 ng, 15-min).

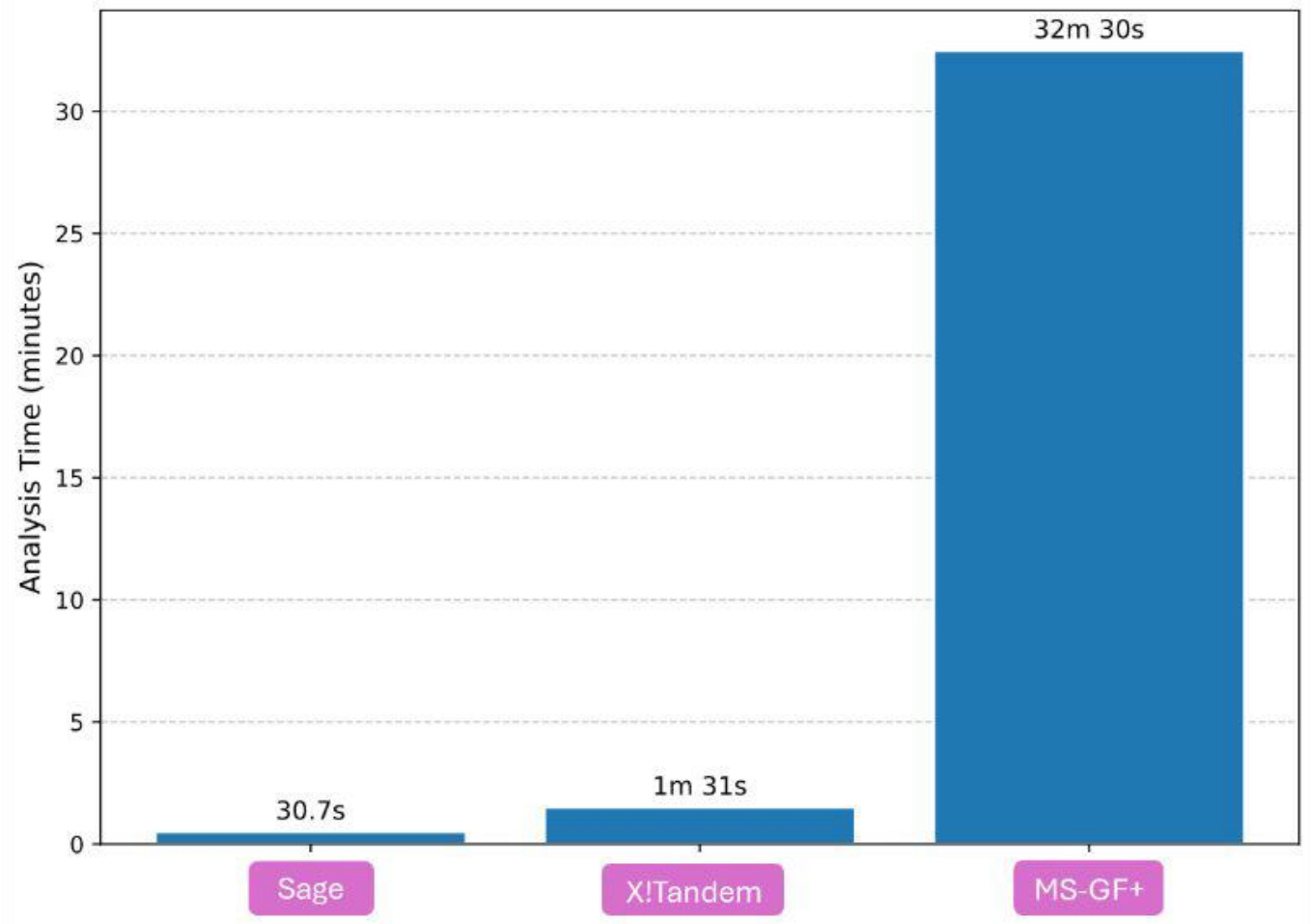

Supplemental Figure 5. Computational performance comparison of peptide search engines. Analysis time for a LC-MS/MS run across three search engines acquired on Q Exactive HF orbitrap platform to show practical advantages of different search engines.

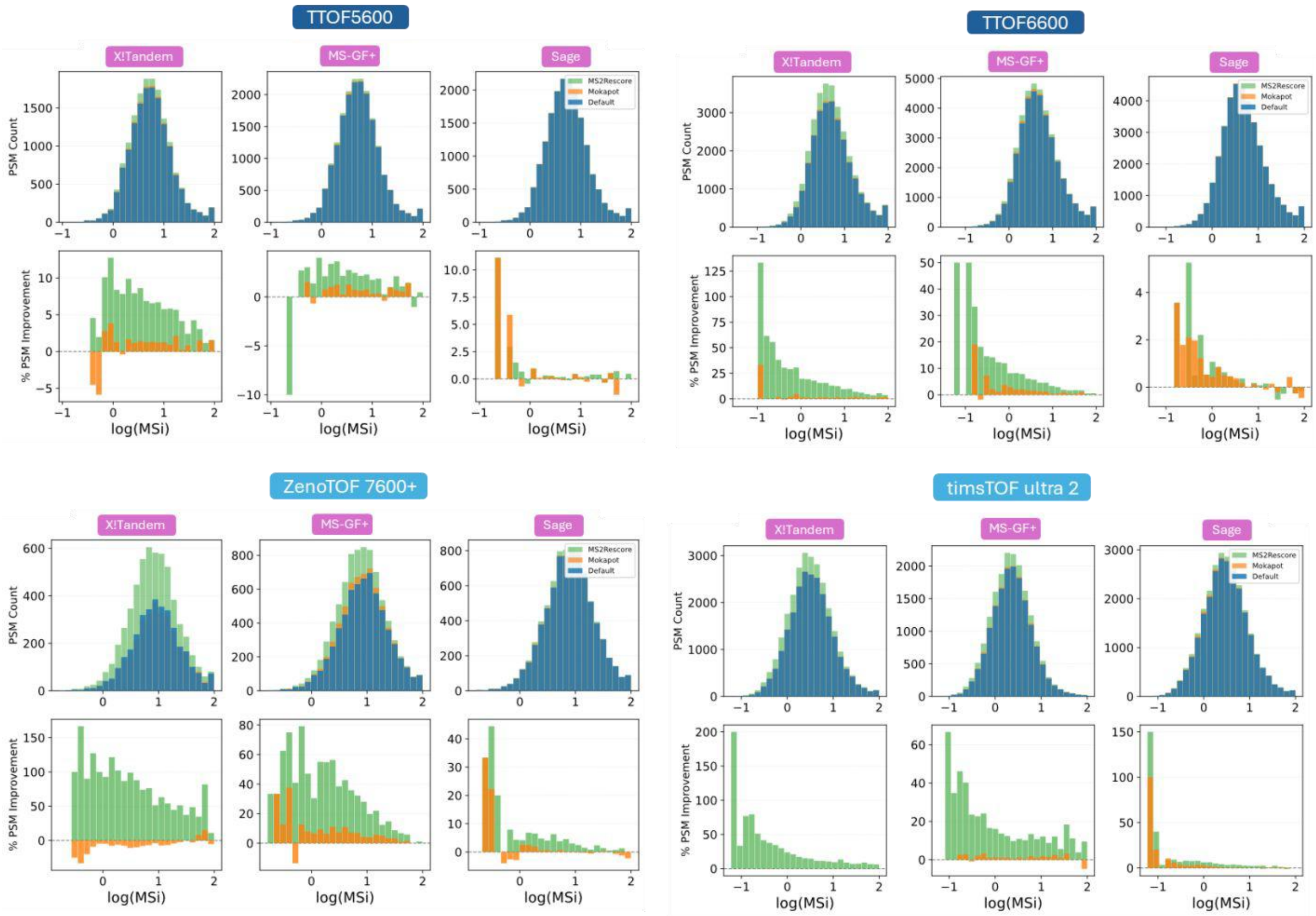

Supplemental Figure 6. Machine learning-based rescoring preferentially recovers low-abundant precursors. Representative examples from four instrument platforms showing distributions of log-transformed normalized precursor intensity (log(MSi)) for baseline (blue), Mokapot (orange), and MS²Rescore (green) conditions (upper panels) and bin-wise percentage improvement in PSM counts relative to baseline (lower panels). Data shown are from the first replicate of condition α for each platform. Results are shown for three search engines (X!Tandem, MS-GF+, Sage).

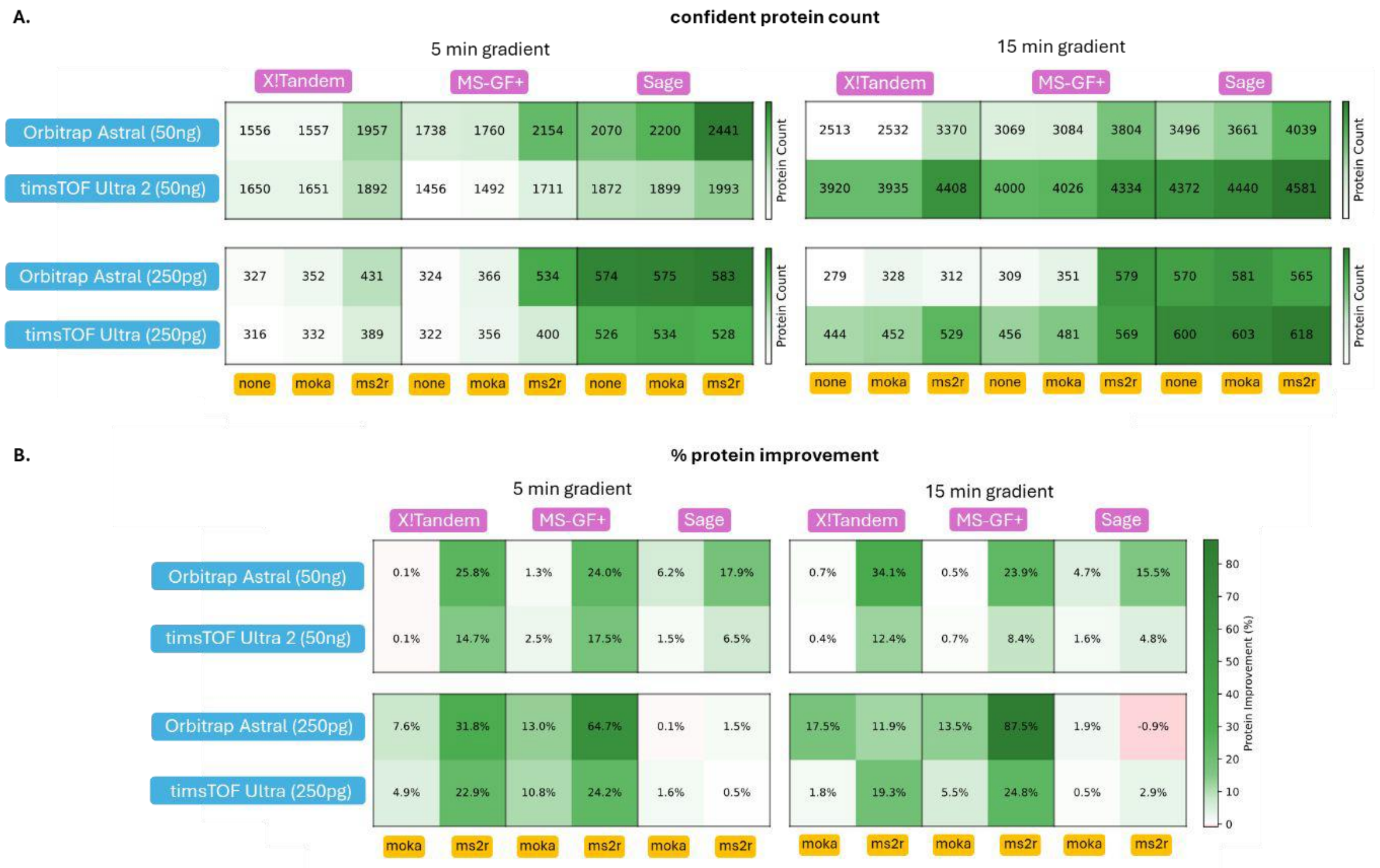

Supplemental Figure 7. Impact of sample load and gradient length on identification performance and rescoring effectiveness across modern instruments. (A) shows absolute protein counts at 1% FDR for Orbitrap Astral and timsTOF Ultra and Ultra 2 across four acquisition conditions varying in sample load (50 ng vs. 250 pg) and gradient length (5 min vs. 15 min). Results are shown for three search engines (X!Tandem, MS-GF+, Sage) and three post-processing approaches (none, Mokapot, MS²Rescore abbreviated as ms2r), (mean of n=6). Color intensity indicates identification count, with darker green representing higher performance. **(B)** shows percentage improvement in protein identifications with rescoring (moka=Mokapot or ms2r=MS²Rescore) relative to baseline (no rescoring) for each acquisition condition. Positive values (green) indicate gains from rescoring; negative values (red) indicate losses. Color scale is consistent across all percentage improvement panels to enable direct comparison of rescoring effectiveness across conditions.

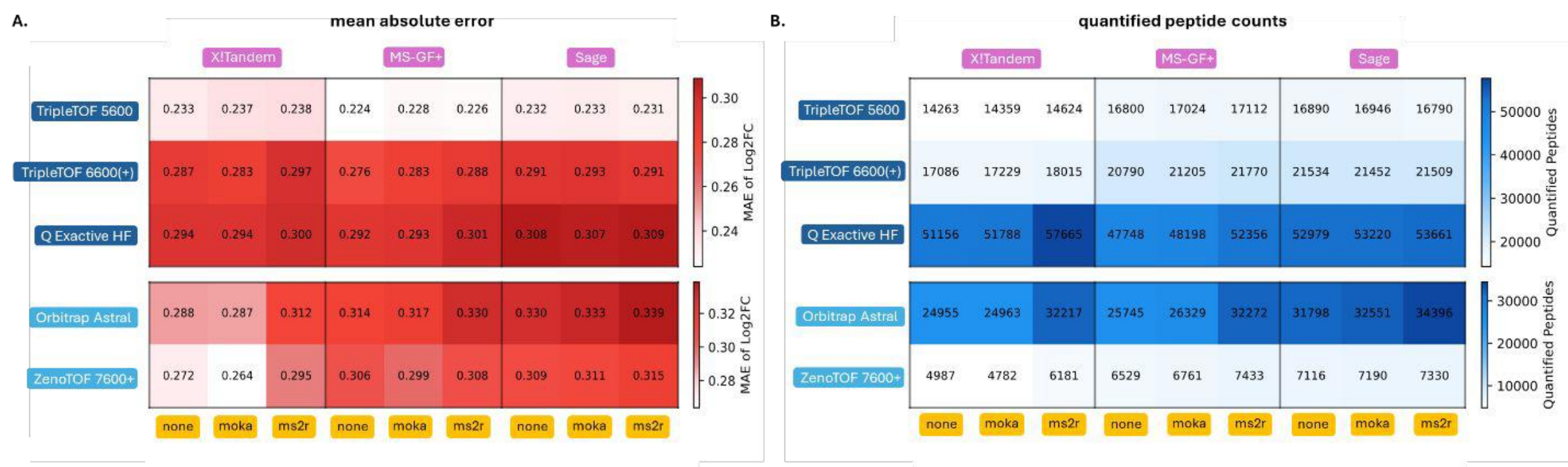

Supplemental Figure 8. Trade-off between identification depth and quantification accuracy. Heatmaps displaying the Mean Absolute Error (MAE) of log2 fold-changes (**A**) and the count of quantified peptides (**B**). The layout of instruments (rows) and software configurations (columns) is identical to Figure 2 (chronological ordering). The horizontal gap separates experiments using high-load/long-gradients (top) from low-load/rapid-gradients (bottom) to prevent confounding comparisons. Darker red indicates higher quantification error; darker blue indicates higher peptide counts.

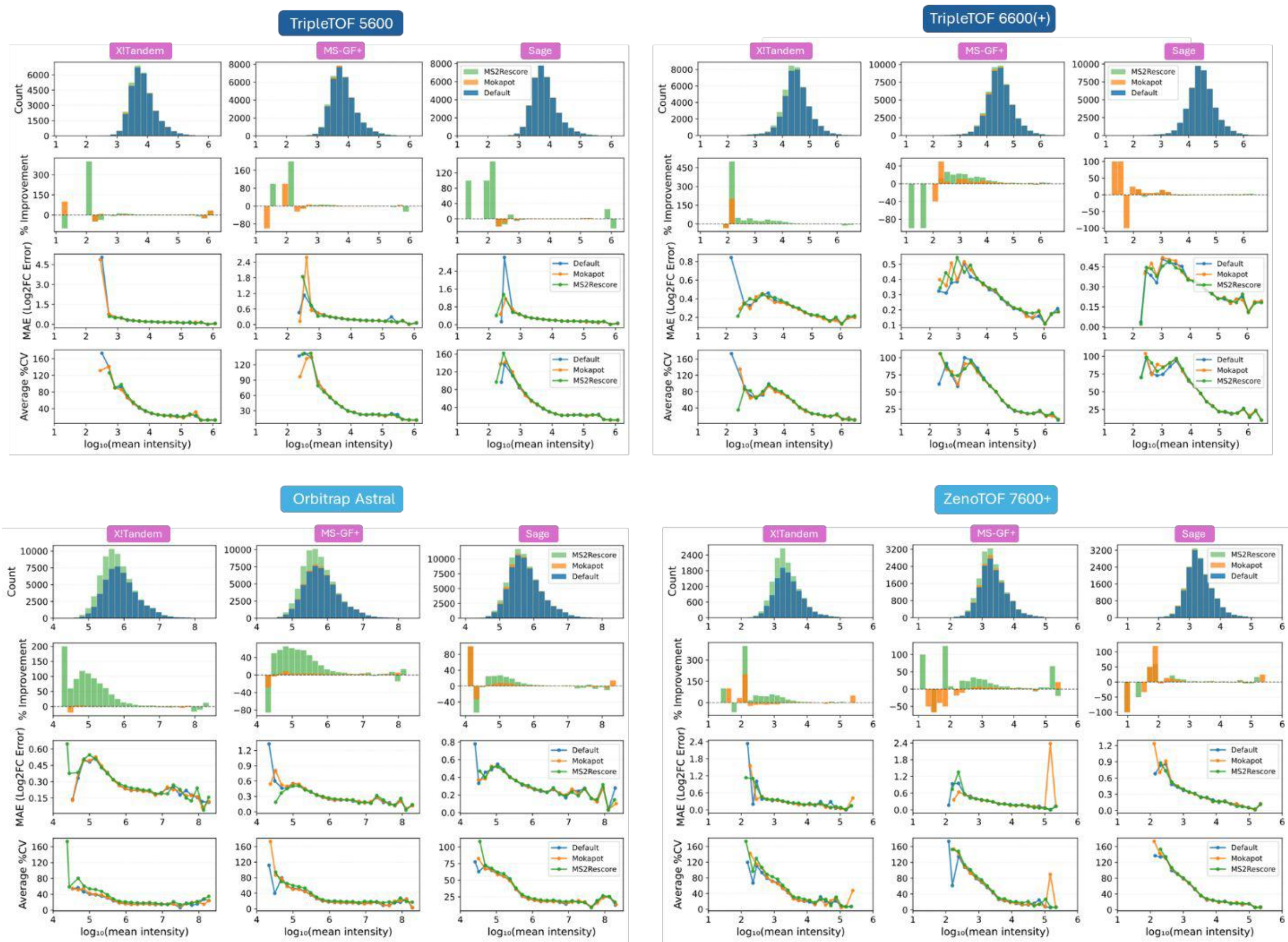

Supplemental Figure 9. Intensity-dependent quantification patterns across mass spectrometry platforms reveal consistent compositional shifts from rescoring methods. Each panel set shows intensity-binned quantification analysis for one representative replicate per instrument and search engine (X!Tandem, MS-GF+, Sage). Within each panel set, four rows display: **(Top)** Distribution of peptide precursor intensities across log-transformed intensity bins for default (blue), Mokapot (green), and MS²Rescore (orange) conditions. **(Second)** Percent improvement in peptide counts relative to baseline, showing rescoring predominantly recovers low-abundance peptides. **(Third)** Mean absolute error of $\log_2$ fold-changes across intensity bins, demonstrating that quantification error increases sharply at low precursor intensities. **(Bottom)** Coefficient of variation across technical replicates as a function of precursor intensity, confirming reduced precision for low-abundance peptides. These patterns are consistent across all platforms, establishing that elevated population-level quantification errors with rescoring result from compositional shifts toward inherently more challenging low-abundance peptides rather than degraded performance on commonly identified species.

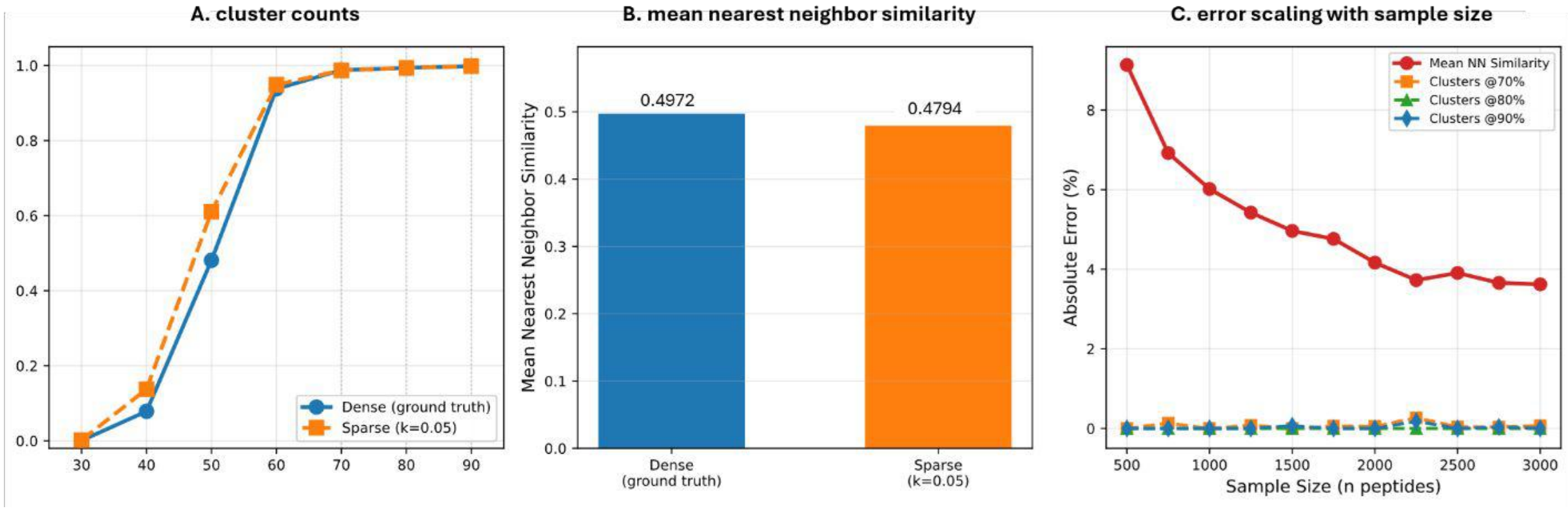

Supplemental Figure 10. Validation of sparse similarity matrix approximation for sequence diversity metrics. To validate our sparse matrix approximation, we compared results against computationally feasible dense matrices calculated on peptide subsets. The approximation introduced negligible error at the 70% similarity threshold, with error only slightly increasing at lower thresholds. Importantly, the error was consistently one-sided—our approach underestimates network connectedness by excluding potential edges—meaning our diversity estimates are conservative. Error decreased with larger subset sizes and remained acceptably small for all thresholds used in our primary analyses. **(A)** Normalized cluster counts across similarity thresholds comparing dense (ground truth) and sparse (k=0.05) matrix approaches. The sparse approximation closely tracks the dense matrix across all thresholds, with slight overestimation at lower thresholds due to missed edges. **(B)** Mean nearest neighbor similarity comparison between dense and sparse approaches, showing minimal deviation (0.4972 vs 0.4794). **(C)** Absolute error of sparse approximation as a function of sample size. Mean nearest neighbor similarity error decreases substantially with increasing peptide counts, while cluster-based metrics at 70%, 80%, and 90% similarity thresholds maintain consistently low error (<1%) across all sample sizes. These validation results confirm that the sparse matrix approach provides reliable diversity estimates while enabling computational tractability for large peptide datasets.

3 Reference

(1) Pedregosa, F.; Varoquaux, G.; Gramfort, A.; Michel, V.; Thirion, B.; Grisel, O.; Blondel, M.; Prettenhofer, P.; Weiss, R.; Dubourg, V.; Vanderplas, J.; Passos, A.; Cournapeau, D.; Brucher, M.; Perrot, M.; Duchesnay, E.; Louppe, G. Scikit-Learn: Machine Learning in Python. *J. Mach. Learn. Res.* **2012**, *12*.
